# Marker-assisted pyramiding of *lycopene-*ε*-cyclase*, *β*-*carotene hydroxylase1* and *opaque2* genes for development of biofortified maize hybrids

**DOI:** 10.1038/s41598-021-92010-8

**Published:** 2021-06-16

**Authors:** Jagveer Singh, Shikha Sharma, Amandeep Kaur, Yogesh Vikal, Amandeep Kaur Cheema, Balraj Kaur Bains, Noorpreet Kaur, Gurjit Kaur Gill, Pawan Kumar Malhotra, Ashok Kumar, Priti Sharma, Vignesh Muthusamy, Amarjeet Kaur, Jasbir Singh Chawla, Firoz Hossain

**Affiliations:** 1 School of Agricultural Biotechnology, Punjab Agricultural University, Ludhiana, India; 2 Department of Plant Breeding and Genetics, Punjab Agricultural University, Ludhiana, India; 3 Department of Plant Breeding and Genetics, Regional Research Station, Gurdaspur, India; 4 Department of Food Science and Technology, Punjab Agricultural University, Ludhiana, India; 5 Division of Genetics, Indian Agricultural Research Institute, New Delhi, India

**Keywords:** Biotechnology, Plant sciences

## Abstract

Malnutrition affects growth and development in humans and causes socio-economic losses. Normal maize is deficient in essential amino acids, lysine and tryptophan; and vitamin-A. Crop biofortification is a sustainable and economical approach to alleviate micronutrient malnutrition. We combined favorable alleles of *crtRB1* and *lcyE* genes into *opaque2* (*o2*)-based four inbreds viz*.* QLM11, QLM12, QLM13, and QLM14 using marker-assisted backcross breeding. These are parents of quality protein maize versions of two elite hybrids viz*.* Buland and PMH1, grown in India. Gene-based SSRs for *o2* and InDel markers for *crtRB1* and *lcyE* were successfully employed for foreground selection in BC_1_F_1_, BC_2_F_1_, and BC_2_F_2_ generations. The recurrent parent genome recovery ranged from 88.9 to 96.0% among introgressed progenies. Kernels of pyramided lines possessed a high concentration of proA (7.14–9.63 ppm), compared to 1.05 to 1.41 ppm in the recurrent parents, while lysine and tryptophan ranged from 0.28–0.44% and 0.07–0.09%, respectively. The reconstituted hybrids (RBuland and RPMH1) showed significant enhancement of endosperm proA (6.97–9.82 ppm), tryptophan (0.07–0.09%), and lysine (0.29–0.43%), while grain yield was at par with their original versions. The dissemination of reconstituted hybrids holds significant promise to alleviate vitamin-A deficiency and protein-energy malnutrition in developing countries.

## Introduction

Maize is treasured as a profitable crop for its productivity and nutritive qualities and is also referred to as “Queen of Cereals”^1^. It is currently cultivated in more than 150 countries with a total global production of 1147.62 million tons from 193.73 million ha land with an average yield of 5922 kg/ha^2^. It provides about 30% of the food calories to more than 4.5 billion people in developing countries and also serves as a major component of animal feed^3^. It is considered as a poor man’s nutria-cereal due to its high protein content, carbohydrates, fats, and few of the vital vitamins and minerals^4^.

The maize kernel constitutes 16.5% of the protein, 3–5% oil, 3% crude fiber, up to 2% soluble sugars, 15% water, and 65–70% starch^5^. The major constituent of endosperm is starch, and about 8–10% is grain protein, of which 60% is composed of prolamins known as zeins^4^. However, zeins are devoid of the essential amino acids, lysine and tryptophan, make maize’s nutritional quality poor. The discovery of classic recessive mutant *opaque2* (*o2*) has improved the grain nutritional value by reducing the synthesis of zein proteins and increases endosperm lysine and tryptophan by about 2-folds^6^. *O2* gene localized on chromosome 7 encodes an endosperm-specific bZIP transcription factor that recognizes *O2* box in the promoters of α- and β-zein genes and regulates their expression. As a result, the mutated *o2* gene has several pleiotropic effects like soft texture, susceptibility to diseases, shriveled kernels, and decline in yield. Later on, breeding *o2* mutants with hard vitreous kernels and retaining the high lysine content has led to the development of Quality Protein Maize (QPM). The nutritional benefits of QPM have been well documented in feeding trials^7,8^. A number of QPM conversion programs have been successfully accomplished^9–12^.

Vitamin-A deficiency (VAD) has emerged as a pressing health issue that affects millions of people from many countries in the world^13^. Maize possesses both proA (α-carotene, β-carotene, and β-cryptoxanthin) and non-proA (lutein and zeaxanthin) carotenoids. Most of the reports concluded that the maize kernels naturally exhibit an appreciable variation in carotenoid levels^14,15^. However, commercially grown yellow varieties in many parts of the world contain less than 1.5 ppm of proA compared to the target level of 15 ppm set by Harvest Plus under the biofortification program initiated by CIMMYT^16^. Optimizing β-carotene accumulation requires enhanced flux to the β-branch of the pathway in combination with limiting hydroxylation of β-carotene to downstream xanthophyll compounds that no longer have provitamin-A activity. The key candidate genes influencing β-carotene concentration are *lycopene ε-cyclase* (*lcyE*) located on chromosome 8 and *β-carotene hydroxylase1* (*crtRB1*) present on chromosome 10 that results in enhancement of proA in maize^14,17,18^. Three functional polymorphisms, 5ˊTE (in the 5ˊ-Untranslated Region), InDel (in the coding region), and 3ˊTE (in the 3ˊ-Untranslated Region) have been identified through association mapping in both genes, and favorable alleles have significantly increased the β-carotene content. Thus, breeding maize for increased levels of proA and essential amino acids will play a crucial role, especially in the developing world to combat vitamin-A deficiency and protein-energy malnutrition (PEM) in achieving nutritional security more holistically^14,17,19^.

The favorable alleles of *crtRB1, lcyE*, and *o2* genes hold an immense perspective for the development of beta-carotene plus QPM enriched lines in an accelerated and resource proficient manner without progeny testing through marker-assisted selection (MAS)^20–22^. It also considerably reduces the breeding cycles required to retain the recurrent parent genome (RPG)^10^. Substantial efforts have been put forward to enhance the proA level in maize^15,23,24^. But a few breeding efforts have been made to combine two traits *i.e.*QPM and beta-carotene^13,21,25^. The present scenario necessitates developing maize genotypes with a combination of QPM and proA so that value-added product reaches poor communities. Buland and PMH1 are the popular maize hybrids released for cultivation in the North-Western Plain Zones of India. Our previous investigation has led to the development of QPM versions viz*.,* BulandQ (QLM11 × QLM12) and PMH1Q (QLM13 × QLM14)^12^. The present study was thus aimed to (i) pyramid the favorable alleles of *crtRB1* and *lcyE* genes into QPM background using marker-assisted backcross breeding (MABB), (ii) evaluate the nutritional quality of the introgressed lines and reconstituted hybrids, and (iii) assess the agronomic performance and yield potential of the MAS-derived genotypes.

## Results

### Parental polymorphism

The recurrent parents (QLM11, QLM12, QLM13, and QLM14) and donor parent (HP467-15) were authenticated for *crtRB1* 3ˊTE *and lcyE* 5ˊTE polymorphism using gene-based markers. All the QLM inbreds revealed 296 bp fragment, while donor inbred showed 543 bp of amplicon for *crtRB1* 3ˊTE locus (Supplementary Fig. S1a). Likewise, all recurrent parents registered 300 bp unfavorable allele whereas donor parent possessed 650 bp favorable allele at *lcyE* 5ˊTE locus (Supplementary Fig. S1b). Also, the recessive *o2* allele was present in all QPM recurrent parents while the donor parent had dominant *O2* allele. The SSR marker *phi057* distinguished between the recurrent parents- QLM12, QLM14 (*o2:* 170 bp) and donor parent- HP467-15 (*O2:* 140 bp) (Supplementary Fig. S1c). While QLM11, QLM13 (*o2:* 145 bp), and donor parent (*O2:* 160 bp) were discriminated for *O2* locus with SSR marker *umc1066* (Supplementary Fig. S1d). All the gene-based markers were inherited co-dominantly, and thus selection of heterozygotes in backcross generations is simple and straightforward. SSR markers spanning all the 10 maize linkage groups were analyzed for parental polymorphism between QLM inbreds and CIMMYT β-carotene donor inbred for identification of polymorphic markers to follow background selection. Of the 324 genome-wide SSR markers genotyped on parental inbreds, 161 (49.69%), 134 (41.35%), 144 (44.44%), and 120 (37.03%) SSR markers showed polymorphism between QLM11, QLM12, QLM13, QLM14, and HP467-15, respectively (Supplementary Table S1; Supplementary Fig. S2). The polymorphic markers identified were then subsequently used in screening backcross progenies for the selection of genotypes with the highest background recovery.

### Marker-assisted selection of *crtRB1*, *lcyE*, and *o2*

**Figure 1:**
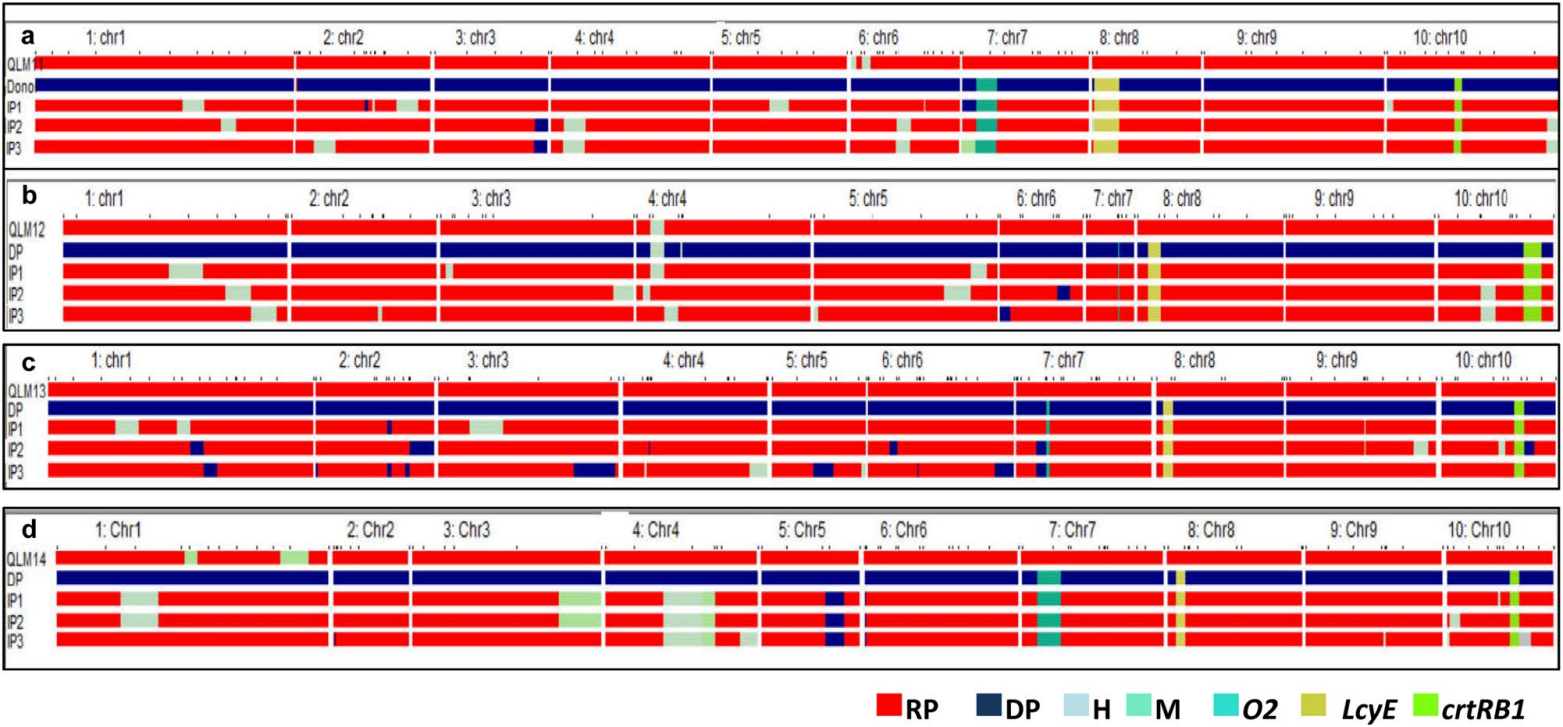
Graphical genotype of selected BC_2_F_1_ progenies across the four crosses. (**a**), QLM11 × HP467-15//QLM11; (**b**), QLM12 × HP467-15//QLM12; (**c**), QLM3 × HP467-15//QLM13; (**d**), QLM14 × HP467-15//QLM14. DP, Donor Parent; IP, Introgressed progeny; Chr, Chromosome.

A total of 390 BC_1_F_1_ progenies of QLM11 × HP467-15/QLM11 were screened with *crtRB1* 3ˊTE marker and 180 were heterozygous for the *crtRB1* allele. These 180 plants were subjected to screening for *lcyE* 5ˊTE marker. A total of 75 plants were identified as heterozygous for both *crtRB1* and *lcyE* genes. Further, these positive plants were analyzed with *o2* specific marker and 35 plants were homozygous for the *o2* allele (Table 1). Similarly, a total of 45, 49, 19 plants were heterozygotes for both *crtRB1* and *lcyE* alleles and homozygous for *o2* allele in QLM12 × HP467-15/QLM12, QLM13 × HP467-15/QLM13, and QLM14 × HP467-15/QLM14 crosses, respectively (Table 1). The BC_1_F_1_ plants were visually assessed for few phenotypic traits (tassel shape, tassel density, anther color, silk color) towards respective recurrent parent type (Supplementary Table S2). Based on visual observations 25, 20, 30, 15 plants of QLM11 × HP467-15/QLM11, QLM12 × HP467-15/QLM12, QLM13 × HP467-15/QLM13, and QLM14 × HP467-15/QLM14 crosses, respectively, were selected for background selection. A total of 100, 90, 121, and 97 SSR markers were employed for background selection in QLM11 × HP467-15/QLM11, QLM12 × HP467-15/QLM12, QLM13 × HP467-15/QLM13, and QLM14 × HP467-15/QLM14 crosses, respectively. The recovery of the recipient genome varied from 70.0 to 92.8% across four crosses in BC_1_F_1_ progenies. The best ones were selected and again backcrossed onto respective recurrent parents to generate BC_2_F_1_ progenies (Table 2). Three ears from each cross were selected based on kernel color and ear shape towards respective recurrent parent to raise BC_2_F_1_ progenies (Supplementary Table S2). Among the BC_2_F_1_ progenies 28, 32, 52, and 43 plants from QLM11 × HP467-15//QLM11, QLM12 × HP467-15//QLM12, QLM3 × HP467-15//QLM13, and QLM14 × HP467-15//QLM14, respectively, were heterozygous for *crtRB1, lcyE* alleles and homozygous for *o2* loci. These foreground positive plants were then again assessed phenotypically on the basis of tassel shape, tassel density, anther color, and silk color. The morphologically selected plants 13, 15, 19, and 17 from each cross of QLM11 × HP467-15//QLM11, QLM12 × HP467-15//QLM12, QLM3 × HP467-15//QLM13, and QLM14 × HP467-15//QLM14, respectively, were subsequently subjected to background selection with respect to only that fraction of region which was heterozygous and not recovered to respective recurrent parent in BC_1_F_1_. The background recovery varied from 88.9% to 96.0% among selected BC_2_F_1_ progenies across the four crosses (Table 2; Fig. 1).

**Table 1:** Segregation pattern of *crtRB1* 3ˊTE, *lcyE* 5ˊTE and *o2* alleles in different backcross and self- generations of the four crosses.

Cross	Generation	N	C + C +	C + C	CC	χ2	*P*-value	N	L + L +	L + L	LL	χ2	*P*-value	N	O + O +	OO	χ^2^	*P*-value
QLM11 × HP467-15	BC_1_F_1_	390	210	180		2.31	0.13 ns	180	105	75		5.00	0.03*	75	40	35	0.33	0.56 ns
	BC_2_F_1_	188	115	73		9.38	0.0022*	73	45	28		3.96	0.05*	28		28		
	BC_2_F_2_	655	231	298	126	38.98	0.00001*	126	35	70	21	4.67	0.10 ns	21		21		
QLM12 × HP467-15	BC_1_F_1_	434	236	197		3.51	0.06 ns	197	102	95		0.25	0.62 ns	95	50	45	0.26	0.61 ns
	BC_2_F_1_	204	134	70		20.08	0.00001*	70	38	32		0.51	0.47 ns	32		32		
	BC_2_F_2_	725	245	325	155	30.10	0.00001*	155	43	82	30	2.70	0.26 ns	30		30		
QLM13 × HP467-15	BC_1_F_1_	486	276	210		8.96	0.0027*	210	100	110		0.48	0.49 ns	110	61	49	1.31	0 .25 ns
	BC_2_F_1_	364	235	129		30.87	0.00001*	129	77	52		4.85	0.03*	52		52		
	BC_2_F_2_	441	132	194	115	7.68	0.0215*	115	29	61	25	0.70	0.70 ns	25		25		
QLM14 × HP467-15	BC_1_F_1_	270	177	93		26.13	0.00001*	93	49	44		0.27	0.60 ns	44	25	19	0.81	0 .36 ns
	BC_2_F_1_	265	170	95		21.23	0.00001*	95	52	43		0.85	0.36 ns	43		43		
	BC_2_F_2_	312	84	157	71	1.10	0.58 ns	71	20	34	17	0.38	0.83 ns	17		17		

*Significant at *P* = 0.05; ns, non-significant; N, No. of plants genotyped; df, degrees of freedom; C + , unfavorable allele of *crtRB1*; C, favorable allele of *crtRB1*; L + , unfavorable allele of *lcyE*; L, favorable allele of *lcyE*; O + , unfavorable allele of *o2*; O, favorable allele of *o2*.

**Table 2:** Recovery of recurrent parent genome (%) in two backcross generations among the best five introgressed progenies of four crosses.

Cross	Plant no	RPG recovery (%) in BC_1_F_1_ generation	Plant no	RPG recovery (%) in BC_2_F_1_ generation
QLM11 × HP467-15	1-10	90.4	1-10-2-3	91.1
	9-10	89.9	1-10-6-4	90.9
	22-6	88.2	22-6-1-6	90.6
	23-2	90.8	23-2-6-7	91.3
	29-8	92.8	23-2-6-9	94.2
QLM12 × HP467-15	15-1	89.9	16-2-3-7	92.5
	16-2	92.4	16-2-5-2	96.0
	14-4	89.8	14-4-7-4	93.8
	18-5	89.7	18-5-7-9	95.6
	18-6	90.0	18-6-7-2	94.4
QLM13 × HP467-15	38-4	85.5	38-4-8-6	91.4
	43-4	81.2	43-4-5-6	88.9
	48-8	82.9	48-8-3-15	90.6
	55-5	84.6	38-4-10-2	93.7
	56-3	83.3	43-4-6-1	93.5
QLM14 × HP467-15	68-6	91.3	78-2-3-4	91.3
	78-2	88.9	78-2-3-8	90.9
	79-8	90.3	79-8-3-7	91.2
	86-10	92.8	89-3-4-11	91.7
	89-3	90.9	89-3-4-8	92.8

**Figure 2:**
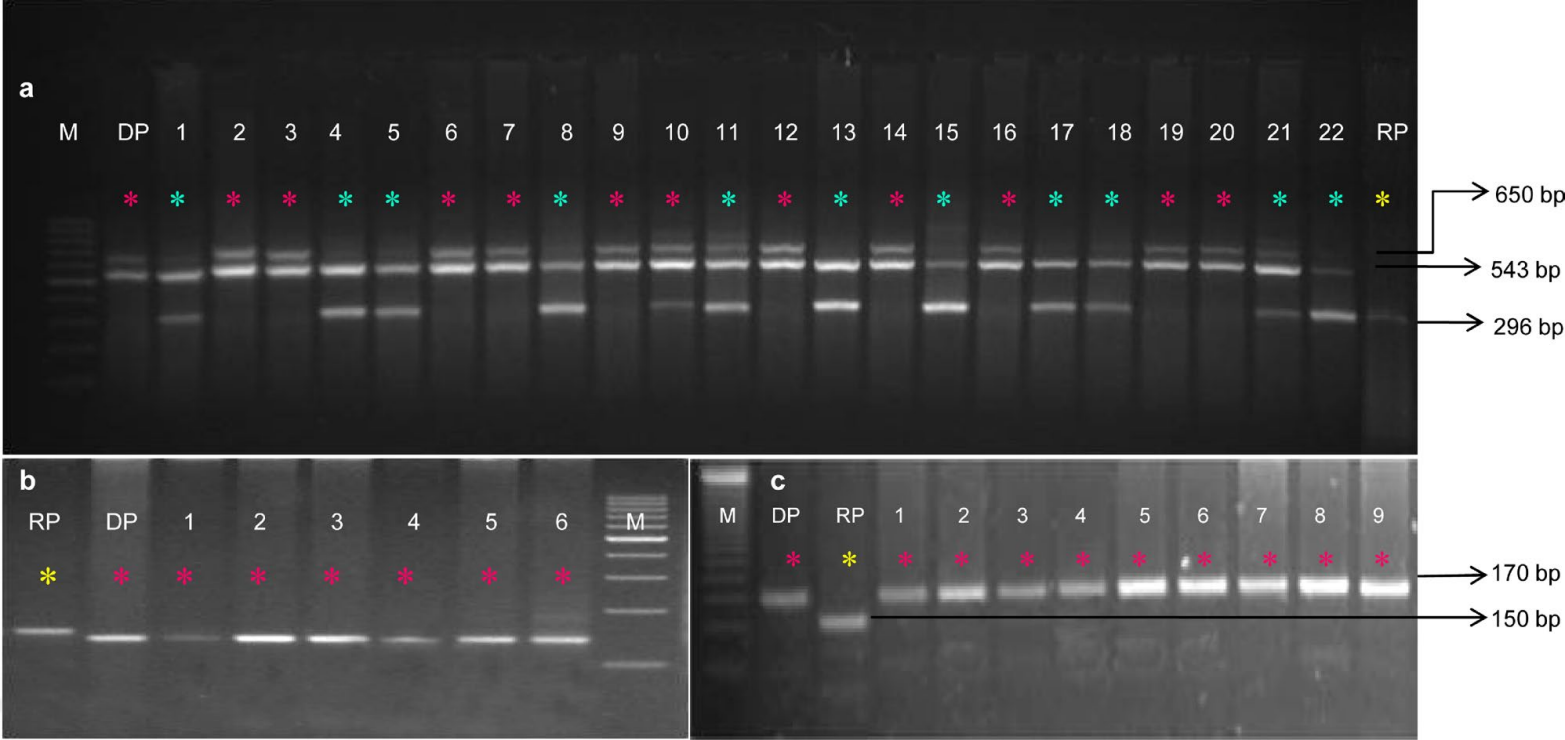
Foreground screening of BC_2_F_2_ progenies using gene specific markers. (**a**), Multiplex PCR for selection of favorable alleles for *crtRB1 and lcyE* genes; (**b**), Amplification profile for selection of *o2* gene using *umc1066* SSR marker; (**c**), Amplification profile for selection of *o2* gene using *phi057* SSR marker. RP: recurrent parent; DP: donor parent; Red star indicates the desirable favorable alleles in homozygous condition; Yellow star indicates homozygous alleles to recurrent parent; Green star indicates both alleles present in heterozygous state.

The introgressed progenies with maximum recovery of the recipient genome and phenotypically similar to the recurrent parent for plant architecture, ear, and grain-related traits were chosen from BC_2_F_1_ progenies in each cross for developing BC_2_F_2_ generation to fix the *crtRB1, lcyE,* and *o2* genes in a homozygous state. A total of 2103 BC_2_F_2_ plants across four crosses were raised from three to five ears/cross and subjected to foreground selection. A total of 21, 30, 25, and 17 plants were homozygous at all three loci in question in BC_2_F_2_ progenies of QLM11 × HP467-15//QLM11, QLM12 × HP467-15//QLM12, QLM3 × HP467-15//QLM13, and QLM14 × HP467-15//QLM14, respectively (Fig. 2). The selected homozygotes were self-pollinated to generate BC_2_F_3_ seeds. The introgressed progenies in the respective backgrounds were grown during the *rainy* season of 2018 and the presence of all favorable alleles was confirmed (Supplementary Fig. S3).

### Agronomic performance of introgressed progenies

**Figure 3:**
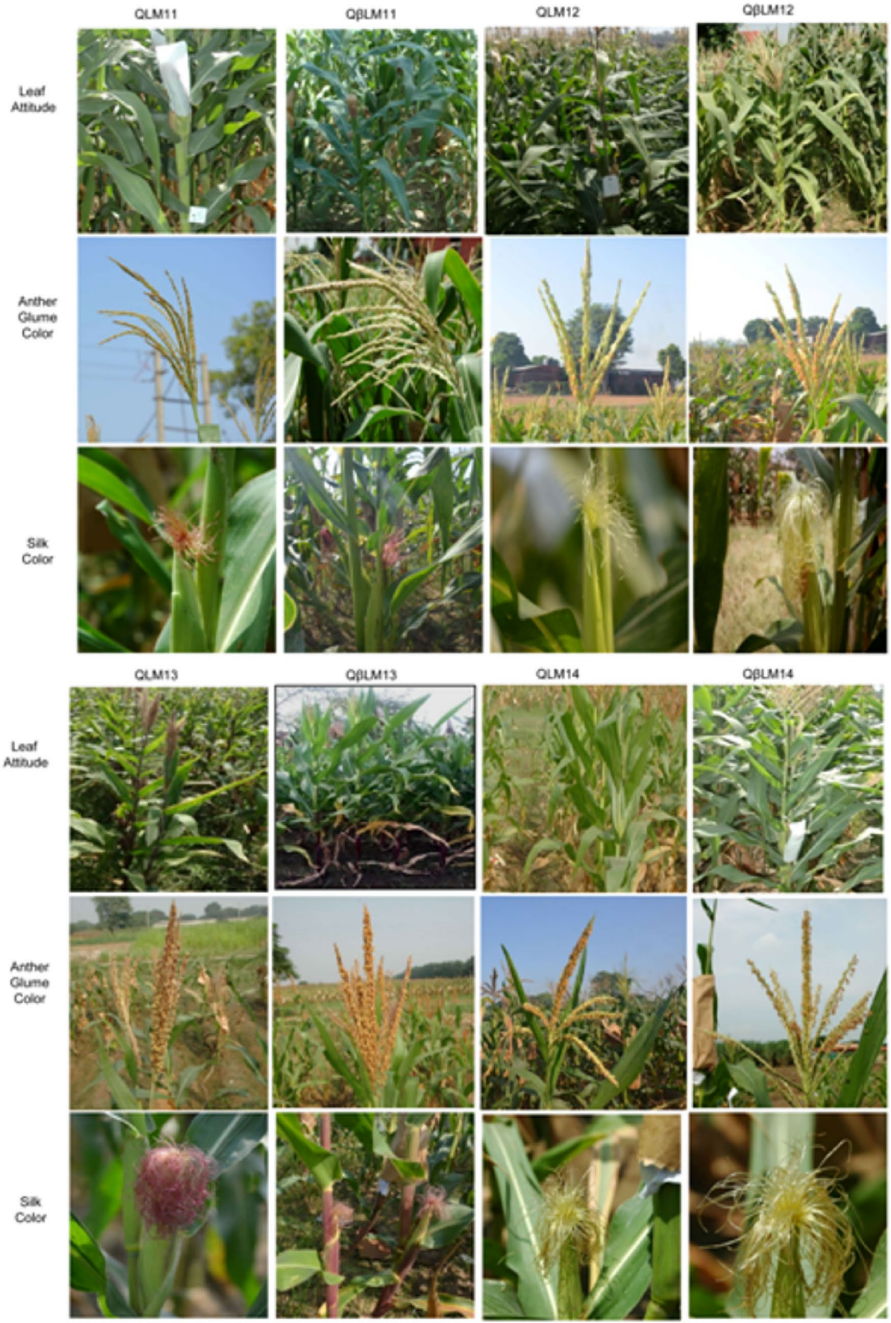
Comparison of morphological traits of the recurrent parents’ vis-à-vis derived introgressed progenies across four crosses.

The selected 18, 22, 17, and 12 BC_2_F_3_ introgressed progenies of QLM11 × HP467-15//QLM11, QLM12 × HP467-15//QLM12, QLM3 × HP467-15//QLM13, and QLM14 × HP467-15//QLM14 crosses, respectively, were evaluated under multi-location trial and revealed high phenotypic similarity with their respective recurrent parents for the various morphological characteristics and grain yield attributing traits (Fig. 3). All the studied traits viz*.*, days to 50% anthesis, days to 50% silking, plant height, cob length, number of kernels per row, and grain yield of the improved versions were at par to original respective inbreds. These introgressed lines exhibited more than 90% similarity to their original inbreds (Table 3; Supplementary Tables S3–S4). The mean yield of QβLM11-1, QβLM12-1, and QβLM13-2 was numerically higher than QLM11, QLM12, and QLM13, respectively. The selection was made on a single plant basis from selected progenies to advance the stable and uniform lines.

**Table 3:** Agronomic performance of selected introgressed progenies vis‐à‐vis respective recurrent parents across the three locations.

Introgressed inbreds	Pedigree	PH (cm)	DTA	DTS	EH (cm)	CG (cm)	CL (cm)	NKRPE	NKPR	GY (kg/ha)
QβLM11-A	QβLM11-22-7-4-8-1-6	164.10	53.78	56.67	90.02	3.69	16.47	15.58	24.33	3511.03
QβLM11-B	QβLM11-22-7-4-8-3-2	165.33	53.28	56.44	92.06	3.07	14.53	14.14	18.40	3168.62
QβLM11-C	QβLM11-22-7-4-8-3-4	161.88	54.22	56.89	90.33	3.51	16.12	17.54	24.68	3021.53
QLM11	-	166.07	53.11	55.56	92.51	3.36	15.59	16.07	19.81	3134.16
QβLM12-A	QβLM12-16-2-7-1-1-1	153.49	53.56	56.78	86.11	3.72	14.65	16.39	19.93	3501.87
QβLM12-B	QβLM12-16-2-10-4-1-4	160.69	54.22	56.56	89.95	3.44	16.43	16.22	22.06	3416.94
QβLM12-C	QβLM12-16-2-7-6-1-6	167.14	52.78	55.33	94.96	3.21	14.96	13.57	21.35	3017.22
QLM12	-	173.16	52.56	55.78	97.17	3.64	14.97	14.34	20.27	3245.36
QβLM13-A	QβLM13-38-4-8-6-4-4	164.42	53.89	57.11	91.82	3.58	16.14	15.34	25.74	3424.66
QβLM13-B	QβLM13-38-4-8-6-5-5	176.78	53.89	56.22	99.83	3.97	17.36	15.12	26.04	3618.23
QβLM13-C	QβLM13-38-4-8-6-6-1	161.49	53.89	56.89	93.47	3.79	16.26	11.81	25.87	3335.54
QLM13	-	164.57	53.89	56.22	93.73	3.76	16.14	15.10	24.32	3196.95
QβLM14-A	QβLM14-79-3-3-5-9-9	166.37	55.22	58.00	94.91	3.44	15.11	14.99	19.76	3323.07
QβLM14-B	QβLM14-79-3-3-9-2-3	164.28	55.22	58.11	95.61	3.59	17.74	16.43	22.11	3423.27
QβLM14-C	QβLM14-89-3-4-8-7-7	166.42	54.89	57.67	95.68	3.07	16.56	16.10	20.47	3426.29
QLM14	–	172.31	55.22	57.44	98.24	3.27	16.14	16.53	20.08	3375.02
SE(d)		6.27	0.75	0.77	3.57	0.13	0.95	0.77	0.98	104.22
CD (0.05%)		17.37	2.07	2.15	9.81	0.37	2.62	2.14	2.71	288.84

PH, plant height; DTA, days to 50% anthesis; DTS, days to 50% silking; EH, ear height; CG, cob girth; CL, cob length; NKRPE, number of kernel rows per ear; NKPR, number of kernels per row; GY, grain yield kg/ha; SE, standard error of difference; CD, critical difference for comparing the averages among sets.

### Biochemical evaluation of introgressed progenies

All of the introgressed lines showed a significant increase in β-carotene over the original inbreds. Lysine and tryptophan contents varied from 0.28 to 0.44% and 0.07 to 0.09%, with an average of 0.34 and 0.08% across crosses (Table 4). The concentration of β-carotene ranged from 4.95 to 7.46 ppm, while β-cryptoxanthin from 3.62 to 4.47 ppm and proA varied from 7.14 to 9.63 ppm. The higher accumulation of proA content may be due to both favorable alleles of *crtRB1* and *lcyE* were introgressed in the background of QPM that has contributed additively. QβLM11-A, QβLM12-C, QβLM13-B, and QβLM14-C ascertained high proA content of 9.28, 9.63, 9.01, and 8.36 ppm, respectively (Table 4). The biochemical parameters were also confirmed in the advanced generations (data not given).

**Table 4:** Biochemical evaluation of selected introgressed progenies with their respective recurrent parents.

Introgressed inbreds	Lysine (%)	Tryptophan (%)	BC (ppm)	BCX (ppm)	proA (ppm)
QβLM11-A	0.298	0.080	7.46	3.65	9.28
QβLM11-B	0.311	0.086	5.70	3.62	7.51
QβLM11-C	0.361	0.066	6.09	4.13	8.16
QLM11	0.345	0.089	1.06	0.70	1.41
QβLM12-A	0.375	0.088	7.01	2.96	8.49
QβLM12-B	0.435	0.075	5.80	4.29	7.94
QβLM12-C	0.333	0.068	7.40	4.46	9.63
QLM12	0.362	0.091	0.75	0.62	1.05
QβLM13-A	0.335	0.090	6.27	3.82	8.18
QβLM13-B	0.383	0.074	6.89	4.25	9.01
QβLM13-C	0.295	0.083	5.39	3.63	7.20
QLM13	0.354	0.086	1.12	0.46	1.35
QβLM14-A	0.362	0.086	4.95	4.39	7.14
QβLM14-B	0.279	0.092	5.87	3.70	7.72
QβLM14-C	0.336	0.079	6.12	4.47	8.36
QLM14	0.321	0.094	1.25	0.22	1.36
SE(d)	0.01	0.00	0.25	0.19	0.21
CD (0.05%)	0.04	0.01	0.69	0.54	0.59

BC, Beta-carotene; BCX, Beta-cryptoxanthin; proA, Provitamin-A; SE, standard error of difference; CD, critical difference for comparing the averages among sets.

### Agronomic performance of reconstituted hybrids

**Figure 4:**
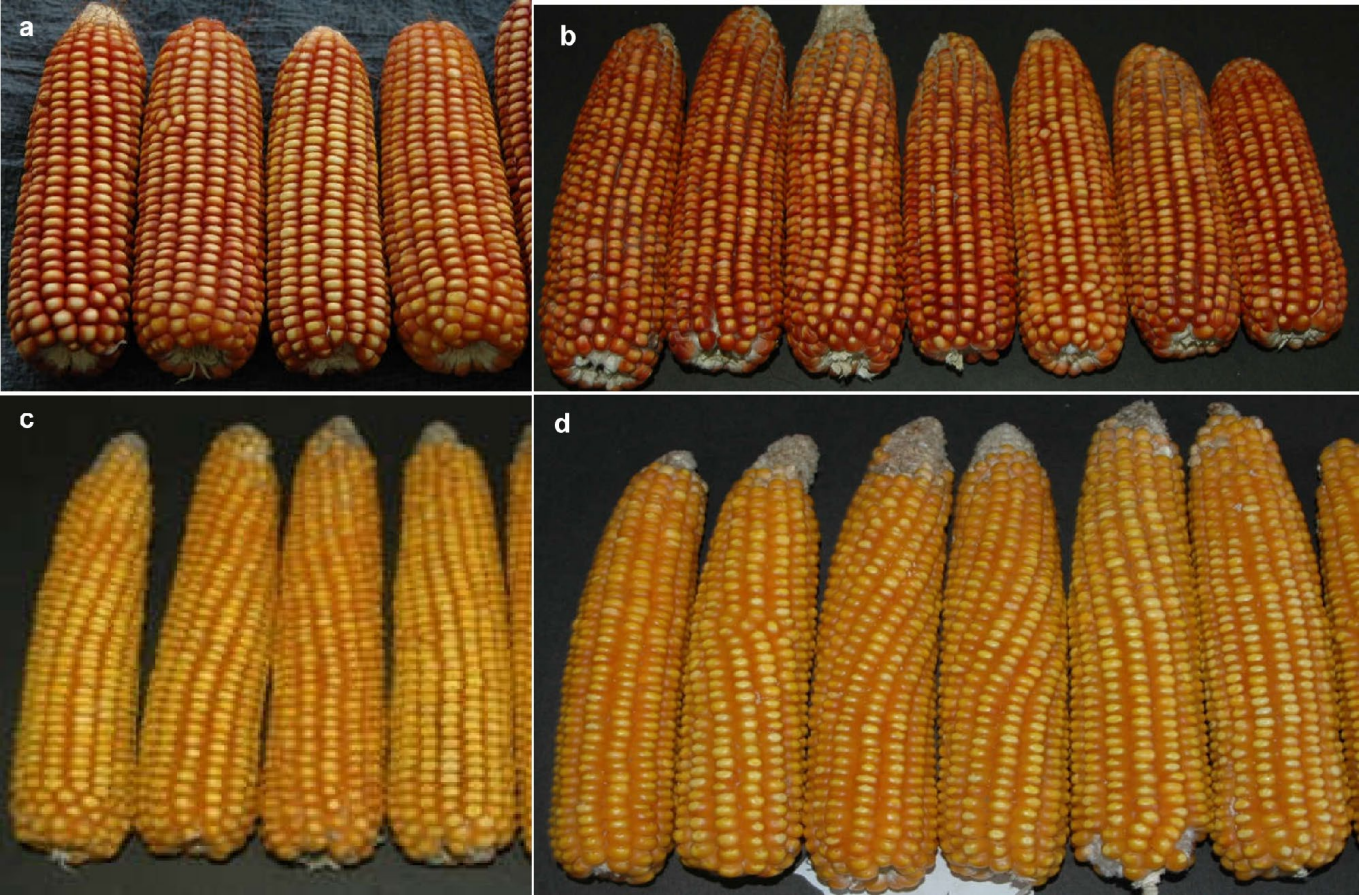
Comparison of original hybrids and reconstituted hybrids with high proA, lysine, and tryptophan for ear and grain characteristics. (**a**), original hybrid Buland; (**b**), reconstituted version of Buland; (**c**), original hybrid PMH1; (**d**), reconstituted version of PMH1.

The improved lines with a similar degree of kernel texture, shape, and color owing to their respective recurrent parents were crossed in original combination to reconstitute QPM + β-carotene enriched maize hybrids. Numerically the reconstituted hybrid versions were at par to their original respective hybrids as well as within same reconstituted hybrid versions with respect to traits like plant height, ear height, cob girt, cob length, and the number of kernels per row (Table 5 and Supplementary Tables S5, S6). The reconstituted hybrids also exhibited a high degree of resemblance for various morphological characters with the respective original versions like late-maturing behavior and medium long ears. The grain characteristics like orange and yellow flint grains of Buland and PMH1, respectively, were retained among the newly reconstituted RBuland and RPMH1 hybrids (Fig. 4).

**Table 5:** Combined analysis of reconstituted hybrids along-with original hybrids across three locations.

Genotype	Pedigree	PH (cm)	DTA	DTS	EH (cm)	CG (cm)	CL (cm)	NKRPE	NKPR	GY (kg/ha)
RBuland-2	QβLM11-A × QβLM12-B	182.60	53.33	55.78	106.41	4.83	17.60	15.99	31.33	6786.44
RBuland-4	QβLM11-A × QβLM12-A	187.41	53.33	55.89	102.57	4.66	18.86	14.94	32.79	7280.04
RBuland-6	QβLM11-B × QβLM12-B	190.05	53.67	56.78	106.77	4.39	16.71	14.89	29.40	6918.63
RBuland-8	QβLM11-C × QβLM12-C	189.26	53.78	56.67	103.02	4.26	15.79	14.17	34.84	6988.30
RBuland-11	QβLM11-C × QβLM12-A	184.46	54.00	56.11	103.48	4.71	20.57	16.51	32.81	7312.80
Buland	LM11 × LM12	183.09	52.67	56.00	102.99	5.10	19.24	16.39	34.42	6919.81
RPMH1-2	QβLM13-C × QβLM14-A	184.69	54.22	56.67	109.46	4.81	20.82	14.67	35.93	8572.82
RPMH1-17	QβLM13-C × QβLM14-A	187.63	54.22	56.67	104.62	4.89	19.49	14.49	32.12	8289.92
RPMH1-24	QβLM13-A × QβLM14-A	193.85	56.33	58.56	109.84	5.05	20.64	16.99	35.02	7736.05
RPMH1-4	QβLM13-A × QβLM14-B	196.75	54.00	57.00	110.57	5.22	23.09	17.36	37.04	8435.94
RPMH1-8	QβLM13-B × QβLM14-B	183.39	53.33	56.44	102.09	4.43	17.47	12.09	30.66	7952.07
PMH1	LM13 × LM14	196.06	55.11	58.22	110.09	5.28	20.54	13.96	35.98	7982.58
HQPM1	-	192.54	53.56	56.22	109.05	4.81	18.73	13.96	35.38	6853.81
SE(d)		8.90	0.97	0.94	4.61	0.22	0.57	0.68	1.22	181.84
CD (0.05%)		24.70	2.70	2.60	12.78	0.06	1.60	1.90	3.40	503.97

PH, plant height; DTA, days to 50% anthesis; DTS, days to silking; EH, ear height; CG, cob girth; CL, cob length; NKRPE, number of kernel rows per ear; NKPR, number of kernels per row; GY, grain yield kg/ha; SE, standard error of difference; CD, critical difference for comparing the averages among sets.

### Biochemical analysis of reconstituted hybrids

**Figure 5:**
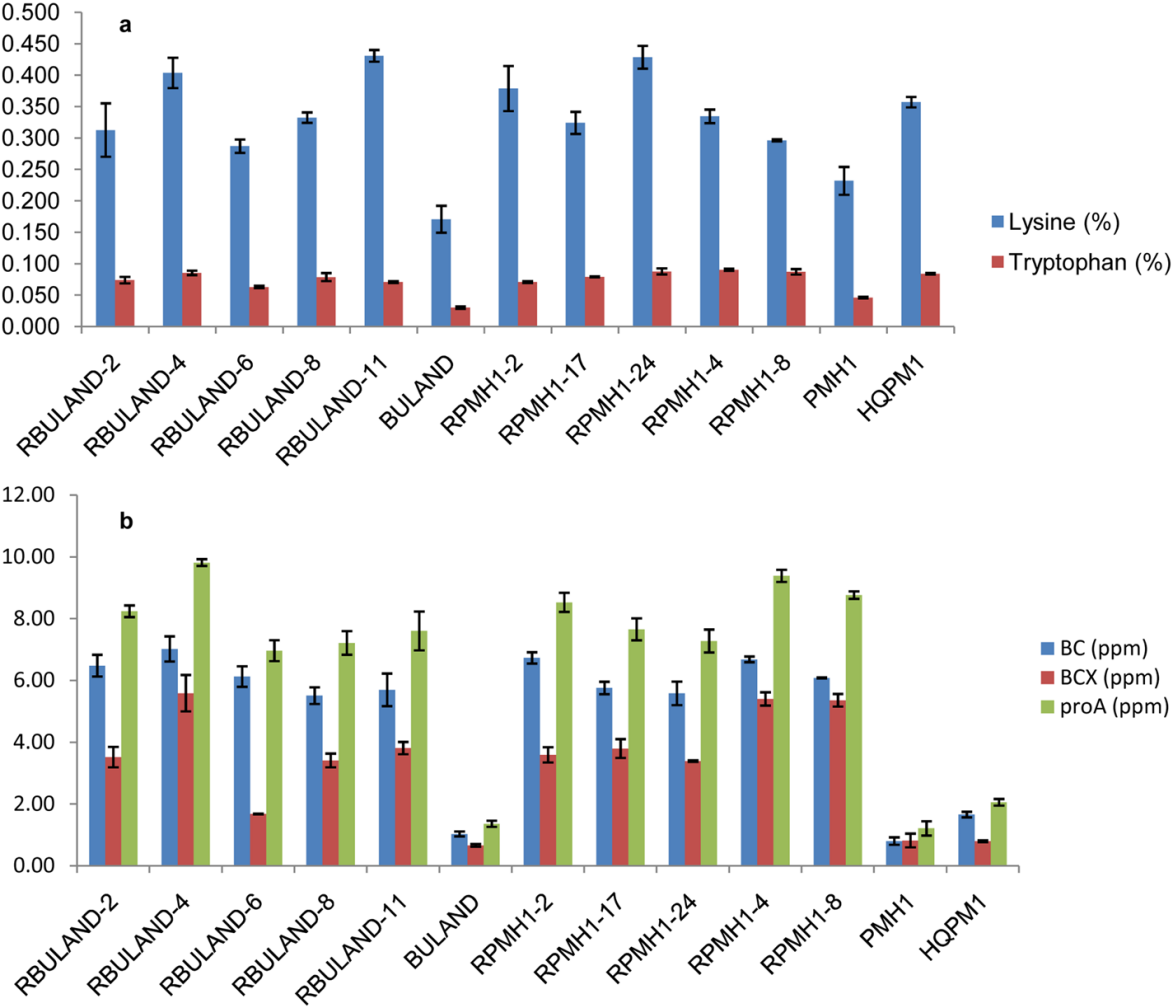
Quality parameters of reconstituted hybrids versions vis‐à‐vis original hybrids. (**a**), Lysine and tryptophan concentration in reconstituted and original hybrids; (**b**), BC, BCX and proA concentration in reconstituted and original hybrids. BC, β-carotene; BCX, β-cryptoxanthin; proA, ProvitaminA; bar indicates standard error.

Tryptophan content ranged from 0.06 to 0.09% in RBuland versions whereas it varied from 0.07 to 0.09% in RPMH1 versions (Fig. 5a). The proA concentration among the RBuland hybrids ranged from 6.97 to 9.82 ppm whereas it varied from 7.28 to 9.39 ppm in RPMH1 hybrids with an increase of 4–6-folds over their original Buland and PMH1 checks (Fig. 5b). The reconstituted hybrid versions had significantly higher β-carotene, β-cryptoxanthin, lysine and tryptophan as compared to their original hybrids (Supplementary Table S7). The reconstituted hybrids of RBuland had Zn content from 34.52 to 36.53 ppm and Fe from 32.13 to 35.94 ppm while Buland possessed 34.82 ppm Zn and 32.55 ppm Fe. Zn and Fe content among RPMH1 hybrid versions varied from 47.87–49.41 ppm and 35.27–38.75 ppm, respectively, whereas PMH1 had 48.30 and 37.10 ppm Zn and Fe, respectively (Supplementary Table S8).

### Chapatti making performance and sensory quality of reconstituted hybrids

The physico-chemical characteristics are an important group as it determines the quality of maize *chapattis*. Statistically significant variations were observed relative to water absorption of flour, roll-ability, and puffing in all reconstituted hybrids versus checks (Table 6). Sensory parameters of *chapatti* prepared from RBuland and RPMH1 hybrids and original hybrids as well as control flour from the market were assigned score based on the Hedonic scale. Sensory scores for appearance varied from 6.80 to 9.73, with higher scores for RBuland-2 *chapattis* as the latter showed an appealing color with light brown spots spread evenly over the surface. Overall acceptability of *chapatti* quality was higher for RPMH1-4 (9.45) and RBuland-8 (8.55) hybrids. Further, the grains of these hybrids were processed to form ready-to-eat finished Massa products using the nixtamalization method, transforming the raw material into food products that are more suitable for commercial trade as they provide convenience and extended shelf life. These shelf-stable products were then used in the production of homemade tortillas which are the main source of carbohydrate and calcium with reduced meal preparation time and convenience.

**Table 6:** *Chapatti* making quality and sensory attributes of reconstituted maize hybrids flour. SE, standard error of difference; CD, critical difference for comparing the averages among sets.

Reconstituted hybrids	Water absorption ml/100 g	Dough handling (Sticky/non sticky)	Roll-ability	Mean sensory scores					
				Puffing	Appearance	Colour	Flavour	Texture	Overall acceptability
RBuland-2	113.44 ± 7.53	Non Sticky	Good	Good	9.73 ± 0.58	8.50 ± 0.58	7.50 ± 0.58	7.53 ± 0.58	8.14 ± 0.58
RBuland-4	111.75 ± 4.74	Non Sticky	Good	Good	7.27 ± 1.16	9.27 ± 1.16	7.83 ± 1.16	10.03 ± 1.16	8.10 ± 1.16
RBuland-6	110.65 ± 5.19	Non Sticky	Good	Good	8.27 ± 1.74	8.67 ± 1.74	7.70 ± 1.73	8.87 ± 1.74	8.13 ± 1.73
RBuland-8	110.48 ± 3.44	Sticky	Very Good	Very Good	7.63 ± 2.32	7.20 ± 2.32	7.03 ± 2.32	9.03 ± 2.31	8.55 ± 2.31
RBuland-11	115.95 ± 4.02	Non Sticky	Good	Good	6.80 ± 2.89	7.47 ± 2.89	5.37 ± 2.89	8.00 ± 2.89	8.01 ± 2.89
Control	112.00 ± 0.46	Non Sticky	Poor	Poor	7.00 ± 3.47	7.00 ± 3.47	8.00 ± 3.47	6.00 ± 3.47	7.00 ± 3.47
Buland	124.08 ± 2.48	Non Sticky	Poor	Poor	8.30 ± 4.05	8.93 ± 4.05	8.27 ± 4.05	9.63 ± 4.05	6.90 ± 4.04
RPMH1-2	117.37 ± 2.67	Non Sticky	Good	Good	8.17 ± 0.52	7.40 ± 0.42	7.60 ± 0.23	8.13 ± 0.54	8.67 ± 0.69
RPMH1-17	101.35 ± 1.36	Non Sticky	Good	Good	8.13 ± 0.51	8.30 ± 0.85	7.23 ± 0.24	8.43 ± 1.17	8.62 ± 0.44
RPMH1-24	118.28 ± 0.01	Non Sticky	Good	Good	8.67 ± 0.01	8.73 ± 0.01	8.10 ± 0.01	8.63 ± 0.01	8.65 ± 0.01
RPMH1-4	114.31 ± 0.02	Sticky	Very Good	Very Good	8.23 ± 0.02	6.97 ± 0.02	6.90 ± 0.03	8.83 ± 0.02	9.45 ± 0.02
RPMH1-8	117.97 ± 0.02	Non Sticky	Good	Good	7.23 ± 0.02	8.30 ± 0.02	7.00 ± 0.02	7.90 ± 0.02	8.61 ± 0.02
Control	112.00 ± 0.46	Non Sticky	Poor	Poor	7.00 ± 0.45	7.00 ± 0.20	8.00 ± 0.72	6.00 ± 0.51	7.00 ± 0.20
PMH1	104.00 ± 0.01	Non Sticky	Fair	Fair	8.00 ± 0.01	8.00 ± 0.01	8.50 ± 0.01	6.00 ± 0.01	7.62 ± 0.05
SE (d)	4.37	**–**	**–**	**–**	0.66	0.54	0.83	0.55	0.5
CD (0.05%)	12.1				1.83	1.51	2.29	1.52	1.38

## Discussion

Globally, QPM varieties were developed to address protein-energy malnutrition problems and significantly benefited the people in underdeveloped nations, particularly in Africa^5^. It has been reported that the QPM diet greatly improved the health of children prone to severe malnutrition^26,27^. However, normal maize cultivars commonly grown by farmers and QPM varieties contain less than 2 mg g^−1^ of proA^15^ which is below the recommended value to meet the daily requirements in a diet^28^. Henceforth, the development and deployment of nutritious biofortified maize with higher protein quality and proA content is one of the most sustainable strategies to tackle “hidden hunger,” more effectively for millions of people who depend upon maize for sustenance. Previous studies have shown that down regulation of *lcyE* reduces the ratio of the α-carotene branch to the β-carotene branch and *crtRB1* favors the accumulation of β-carotene over that of β-cryptoxanthin in the carotenoid biosynthetic pathway, leading to an increase in levels of provitamin-A^14,17^. Four natural *lcyE* polymorphisms (*lcyE* 5ˊTE, *lcyE* SNP216, *lcyE* SNP2238 and *lcyE* 3ˊInDel) and three *crtRB1* polymorphisms (*crtRB1* 5ˊTE, *crtRB1*InDel4 and *crtRB1* 3ˊTE) were identified. The favorable allele of *lcyE* 5ˊTE and *crtRB1* 3ˊTE causes a significant increase in provitamin-A in the endosperm^14,16–18^. Therefore these two loci (*lcy* 5ˊTE and *crtRB1* 3ˊTE) were targeted to introgress into QPM version of elite maize inbreds using MABB.

We employed co-dominant gene specific markers that clearly distinguished all the four recurrent parents (QLM11, QLM12, QLM13, and QLM14) from the donor line HP467-15. The results were consistent with other studies regarding allele size for each marker^10,13,16,21,25^. The foreground selection of target genes in each backcross and selfed generation facilitated the identification of desirable genotypes at the early stage of plant growth. In the BC_1_F_1_ generation, we selected plants that were homozygous for *o2* allele but heterozygous for *crtRB1* and *lcyE* alleles. Therefore, the selected progenies were fixed for *o2* allele and further no segregation occurred for *o2* allele in the subsequent generations. These results are in accordance with earlier reports^13,18,21,29–31^.

It has been observed that during foreground selection in different generations *crtRB1* gene showed segregation distortion across the crosses. Significant segregation distortion (SD) for *crtRB1* was also documented in other studies^13,16,21^. On the contrary, *lcyE* and *o2* genes segregated as per Mendelian inheritance^10–12,25^. We performed simultaneously background selection for recovery of the parental genome using 80–100 genome-wide SSR markers for the selection of desirable genotype in each cross. Background analysis revealed 88.9–96.0% recovery of recurrent parent genome. This enabled us in precise selection of the foreground positive progenies possessing high RPG^10,11,13,16,21,32^. The main emphasis of background selection was to recover the maximum proportion of recurrent parent genome at non-target loci using DNA markers distributed evenly on the genome in an accelerated manner^12^. The identified individuals with a range from low to high RPG content in BC_1_ progenies revealed unbiased sampling and marker data points. Further high RPG recovery in BC_2_ progenies demonstrated that two rounds of background selection resulted in selections of desirable genotypes. Servin and Hospital^33^ illustrated that the use of either a few optimally placed markers or more sub-optimally placed markers can efficiently control large chromosomal regions and leads to better control of the return to the recipient genome. Thus, the criteria of selecting optimal positioning of markers in the present study resulted in maximization of the expected proportion.

Phenotypic evaluation and selection was done in the genotypically identified BC_2_F_3_ improved progenies having *crtRB1, lcyE*, and *o2* genes in homozygous condition. The MAS-derived pyramided lines with high RPG also exhibited a high degree of resemblance with their corresponding recurrent parent for plant architecture, ear type, and grain characteristics as evident from our results and previous studies^10,16,34^. Similarly, reconstituted hybrid (RBuland and RPMH1) versions were at par to checks and original hybrids in grain yield and other attributing traits. This high degree of phenotypic similarity among the reconstituted hybrids is also ascribed to high RPG recovery of the introgressed progenies^10–12,16,25^.

The proA content of the improved lines was increased from 4–8-folds when compared to their original QPM parents while lysine and tryptophan content showed an increase of 1.38–2.50 and 1.68–2.86-folds, respectively across the crosses over the normal parental inbreds. The reconstituted hybrids had 4–6-folds higher proA while 1.40–2.50 and 1.54–2.83-folds increase in lysine and tryptophan over their original versions*.* The variation for lysine and tryptophan among improved progenies could be attributed to the presence of amino acid modifies which varied with different genetic backgrounds^11,12^. Similar results were obtained by previous studies^10,25,35–38^. It was observed that the QPM introgressed inbreds used in the present study possessed comparatively more tryptophan content that might be due to the fact of genetic interaction of QPM inbred with non-QPM β-carotene donor inbred. Also, differences for β-carotene content among the improved lines and reconstituted hybrid versions were due to other genetic loci like *crtRB3*, *CCD1*, and *ZEP1* in β-carotene synthesis pathway apart from favorable alleles of *crtRB1* and *lcyE* genes that might have contributed to the increase of proA^39–43^. The retention of high proA even after four months of storage was observed. It might be due to the presence of favorable alleles for higher retention of proA^44^. Studies on retention of proA during different storage periods showed that degradation of proA occurs at the first three months of storage and gradually stabilizes after six months (4–6 months)^44^. It suggests that *crtRB1* and *lcyE* -based genotypes would still hold immense significance nutritionally over normal maize. The advantage of stacking favorable alleles of *crtRB1* and *lcyE* genes for proA over as single effects has been reported in a number of studies^45–47^. The micronutrient concentrations in the reconstituted hybrids were at par to original hybrids that could be due to high background recovery of introgressed progenies. Our study revealed variability for Fe and Zn between RBuland and RPMH1 indicating the accessibility of wider genetic variation to be utilized for the genetic improvement of kernel micronutrient traits in maize. Previous studies also documented considerable variations for the kernel Fe and Zn concentrations among maize genotypes^48–51^.

It was observed that combined quality parameters of QPM and proA affected flour color, flavor, the texture of *chapatti* but all the values were in acceptable range among versions of reconstituted hybrids as compared to original hybrids. Thus, *chapattis* from reconstituted hybrids have QPM addition with β-carotene and can be considered acceptable to avail nutritional, phytochemical, and health benefits of maize. These reconstituted hybrids when subjected to nixtamalization resulted in Massa with higher nutritional value with value addition of QPM and proA compared to the original hybrids. Similar studies reported that nixtamalized maize has a higher nutritional value (increased bioavailability of niacin, improved protein quality, increased calcium) with reduced mycotoxins content^52–54^. Nutritionally RBuland-4 and RPMH1-4 were better as compared to other versions while overall acceptability as *chapatti* quality was for RBuland-8 and RPMH1-4 versions. Also, the yield of RPMH1-4 was higher whereas yields of both RBuland-4 and RBuland-11 were comparable to each other as well as both outperformed at all locations as compared to the original checks. Overall, RBuland-4 and RPMH1-4 are selected for further seed multiplication and commercialization.

## Conclusions

We report here the successful stacking of *o2*, *crtRB1*, and *lcyE* genes into the background of four QPM inbreds and developed nutritionally enriched hybrids using MABB. The dual (MAS and phenotypic) selection approach provided an opportunity to unite the desirable agronomic traits with enriched nutritional traits in an accelerated manner. Thus, in the near future, the reconstituted hybrids could likely be grown in India without any difference in grain yield and to greater gain in terms of grain nutrition compared with their originals. The newly developed β-carotene enriched lines in the QPM background and their reconstituted hybrids are value-added products that could serve as pre-breeding material for elite line conversion. The clinical trial of nutritionally enriched hybrids in collaboration with a hospital could also be tested on under-nutrition people at a small scale.

## Materials and methods

The present study was conducted at Punjab Agricultural University (PAU), Ludhiana and ICAR-Indian Agricultural Research Institute (IARI), New Delhi, India during the years from 2015 to 2019 with relevant institutional guidelines and legislation. Necessary permission was obtained from the institute for the collection of plant material.

### Plant materials

The investigational material consisted of four elite QPM maize inbreds viz*.* QLM11, QLM12, QLM13, and QLM14. These QPM inbreds were developed by introgression of *o2* gene from different QPM donors to locally adapted inbreds, LM11, LM12, LM13, and LM14 at PAU, Ludhiana^12^. These are the parental inbreds of single cross QPM version hybrids of BulandQ (QLM11 × QLM12) and PMH1Q (QLM13 × QLM14). QLM11 had cylindrical ears with orange-red round and bold kernels, whereas QLM12 possessed small ears with pointed dull orange kernels. Similarly, QLM13 exhibited medium conical-cylindrical ears with bold round dull yellow kernels, while QLM14 also had conical-cylindrical ears having yellow kernels. Likewise, leaf attitude was semi-dropping and dropping in QLM11 and QLM12; and semi-erect in QLM13 and QLM14. The other phenotypic descriptors (tassel shape, tassel density, anther glume color, anther color, and silk color) of QLM inbreds are enlisted in Table S2. However, β-carotene in the kernels of QPM inbreds and their hybrids is low; hence were targeted for β-carotene enrichment. HP467-15 inbred (CIMMYT, Mexico) was used as a pollen contributor for the introgression of high β-carotene content into the QPM genetic background of each recurrent parent. The pedigree information of recurrent parents and their tryptophan content is given in Supplementary Table S9.

### Marker-assisted backcross breeding for development of improved progenies and hybrids

**Figure 6:**
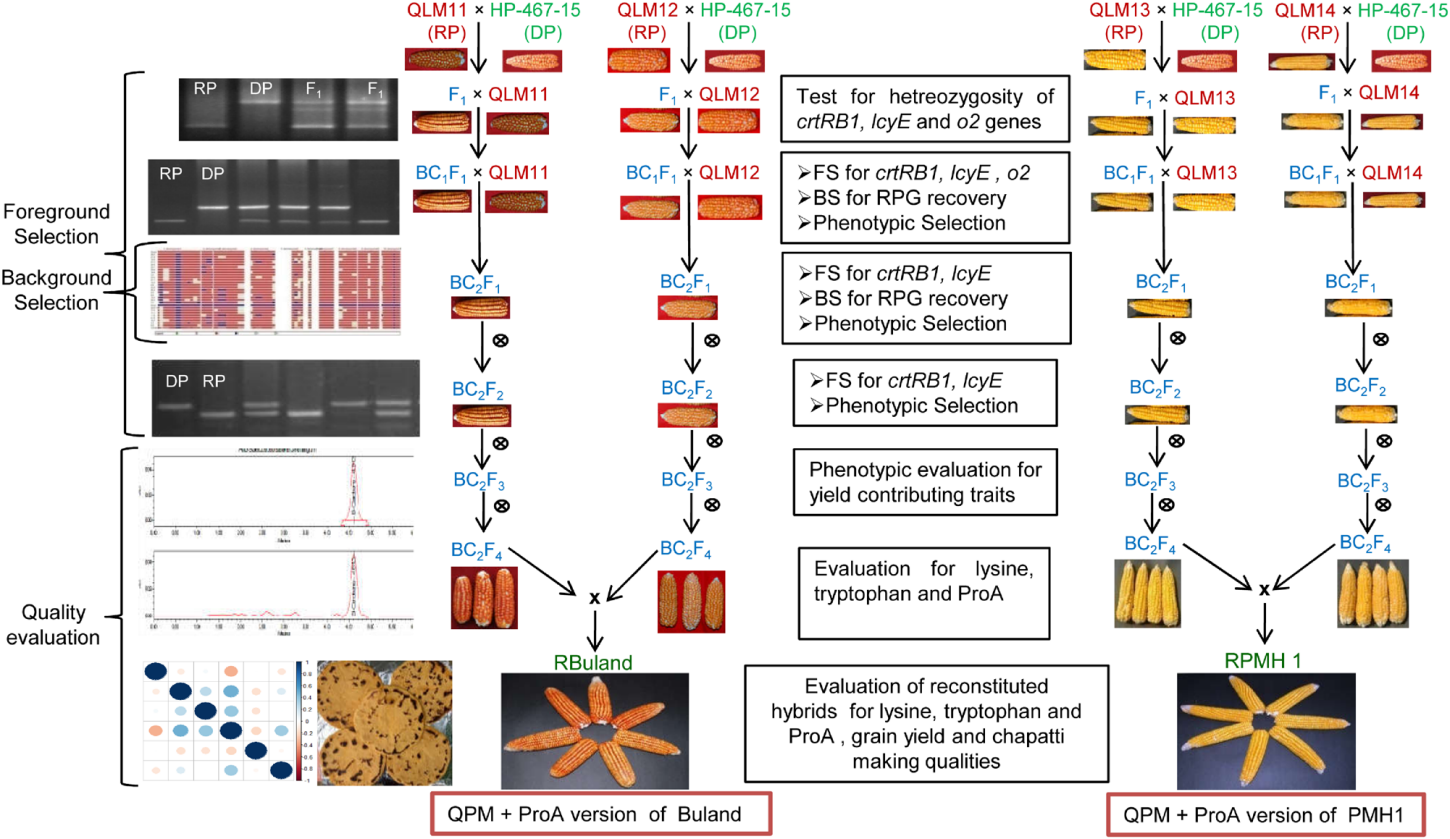
Marker-assisted breeding scheme for the development of *crtRB1, lcyE* introgressed lines in background of four QPM inbreds and reconstitution of original hybrids versions. RP: recurrent parent; DP: donor parent; A: homozygous allele to donor parent; H: heterozygous allele; FS: foreground selection; BS: background selection.

The crossing scheme followed for the reconstitution of nutritionally enriched hybrids and details of population development is represented in Fig. 6 and Supplementary Table S10. The population development involved two parallel crossing schemes. Each QLM inbred was crossed to HP467-15 at ICAR- IARI, New Delhi during the *rainy* season (July–October) 2015. The F_1_s were raised at Punjab Agricultural University (PAU), Ludhiana during the *spring* season (February-May 2016), and the hybridity of F_1_s was confirmed by using target gene-specific markers. The F_1_s of each cross were backcrossed twice to develop BC_2_F_1_ progenies during *rainy* 2016 and *spring* season of 2017. The selected BC_2_F_1_ plants were selfed and advanced to BC_2_F_2,_ progenies that were grown during *rainy* 2017. The homozygous progenies for target loci were selected and selfed to generate BC_2_F_3_ and BC_2_F_4_ progenies during *rainy* 2018 and *spring* 2019. Line conversion for stacking of genes in recurrent background involved different selection indices- foreground, background, phenotypic and biochemical at the appropriate step as specified in the crossing scheme. The promising converted lines were crossed in original hybrid combination to reconstitute biofortified maize hybrids, Buland and PMH1 during *spring* season 2019 and were evaluated during *rainy* season 2019.

### Genomic DNA isolation and PCR analysis

Genomic DNA was extracted following modified CTAB protocol from two-week old seedlings^55^. The DNA quality and quantity was checked by 0.8% agarose gel. Polymerase chain reaction was performed using functional markers for both the *crtRB1* 3ˊTE and *lcyE* 5ˊTE favorable alleles^16,47^. The PCR amplification was performed in a Veriti 96 well (Applied Biosciences, Invitrogen, UK) and GeneAmp PCR system 9700 (Applied Biosystems by Thermo Fisher Scientific) thermal cyclers. The amplification of *lcyE* 5ˊTE was executed by a “touch-down” profile of initial denaturation at 95 °C for 5 min, followed by 16 cycles of denaturation at 95 °C for 45 s, annealing at 58 °C for 45 s and extension at 72 °C for 45 s with each cycle decrease in annealing temperature by 0.5 °C, then 20 cycles were profiled at 95 °C for 45 s, annealing at 56 °C for 45 s and extension at 72 °C for 1 min and a final extension at 72 °C for 10 min. The amplification for *crtRB1* 3ˊTE was achieved using a thermal profile of initial denaturation at 94 °C for 4 min, followed by 25 cycles of denaturation at 94 °C for 1 min, annealing at 53.5ºC for 1.30 min and extension at 72 °C for 1.30 min, and a final extension at 72 °C for 7 min. Multiplex amplification for *crtRB1* 3ˊTE and *lcyE* 5ˊTE was also standardized using a thermal profile of initial denaturation at 95 °C for 10 min, followed by 35 cycles of denaturation at 94 °C for 50 s, annealing at 59 °C for 50 s and extension at 72 °C for 55 s, and a final extension at 72 °C for 8 min. The amplified product was kept on hold at 4 °C after the completion of the thermal profile. SSR gene-based markers (*umc 1066* and *phi 057*) were deployed for the selection of *o2* gene. The PCR profile for amplification of *o2* gene and genome-wide SSR markers was performed using the protocol of Kaur et al.^12^. A negative control (without template DNA) was included in each plate during every amplification reaction. Amplicons were resolved and analyzed using 3.5% Metaphor agarose gel electrophoresis at 120 V for 3-5 h.

### Foreground selection for target genes

Selection for target alleles was performed in both backcrossed populations (BC_1_F_1_ and BC_2_F_1_) using *crtRB1* 3ˊTE*, lcyE* 5ˊTE, and *o2* gene specific markers. Plants having *crtRB1* 3ˊTE and *lcyE* 5ˊTE alleles in heterozygous form were further directed for the selection of *o2* allele with *phi057* and *umc1066* markers in QLM12, QLM14, and QLM11, QLM13 crosses, respectively. The plants having all three genes were subsequently selfed to generate the BC_2_F_2_ progenies. The plants with all three genes in a homozygous state were selected in BC_2_F_2_ progenies across four crosses and subsequently advanced to BC_2_F_3_ progenies. The segregation pattern of each marker locus in each generation was checked by using standard Chi-square analysis for the goodness of fit.

### Background selection for recurrent parent genome

A total of 324 SSR markers spanning all the 10 chromosomes of maize genome were surveyed for polymorphism between each recurrent and donor parent. The coverage of SSR markers per chromosome from each chromosomal bin varied from 14 to 55 markers (Supplementary Table S11). The sequences of the SSR primers were retrieved from maize genome database (www.maizegdb.org) and custom synthesized (IDT, USA). The polymorphic markers were surveyed in BC_1_F_1_ progenies of each cross involving QLM11, QLM12, QLM13, and QLM14 as recurrent parents respectively, for selecting the individuals having high background coverage. The background selection was again preceded in BC_2_F_1_ progenies across crosses for those genomic regions which were not recovered in selected BC_1_F_1_ plants. The amplicon of each marker was registered as ‘A’ for the recurrent parent allele and ‘B’ for the donor allele while ‘H’ for both the alleles. Graphical genotyping (GGT) analysis was carried out to analyze the introgression from donor parent on the individual chromosome using GGT software^56^. The % recurrent parent genome (RPG) recovery was calculated as the ratio of the number of SSRs showing recurrent parent allele in a homozygous state to the total number of polymorphic SSRs applied for background selection.

### Morphological characterization of introgressed progenies

Improved BC_2_F_3_ progenies of each cross along with their original parents were evaluated for phenotypic characteristics and grain yield attributing traits during *rainy* season 2018 at three locations viz*.* (i) Punjab Agricultural University (PAU), Ludhiana, (ii) Regional Research Station (PAU) Gurdaspur and (iii) IARI, New Delhi. Each progeny was raised in two replications in a randomized complete block design (RBCD) and two rows/replication of 3 m row length was grown with a plant-to-plant distance of 20 cm and row to row distance of 60 cm. Standard agronomic practices were followed for raising the crop at each experimental site. The data was recorded for various agronomic characters such as plant height (cm), days to anthesis (days), days to silking (days), cob length (cm), cob girth (cm), number of kernel rows/cob, number of kernels/row and grain yield (Kg/ha). Data were recorded on daily visual observations during the flowering period. Anther glume color, anther color, silk color, tassel shape, tassel density (low to high), and kernel color was documented on a visual basis. Data was subjected to analysis of variance (ANOVA) to determine the significant differences among treatments using SAS (9.4 version) computer software.

### Estimation of proA, lysine, and tryptophan contents

The quality parameters were estimated from BC_2_F_4_ kernels of selected lines across the crosses after 4 months of storage. The procedures specified by Vignesh et al.^29^ were followed for extraction of β-carotene (BC) and β-cryptoxanthin (BCX). BC and BCX were estimated using Dionex Ultimate 3000 UHPLC System (Ultra High-Performance Liquid Chromatography; Thermo Scientific, Massachusetts, USA). The proA concentration (ppm on a dry weight basis) was calculated as the sum of BC plus half the BCX concentration^18^. Lysine and tryptophan content in the endosperm was estimated using Dionex Ultimate 3000 UHPLC system according to the procedure described by Sarika et al.^38^. Each parameter from each sample was estimated from two technical replications. The data generated were analyzed to calculate the standard error of the mean and CD (critical difference) value for comparing the averages among sets.

### Reconstitution of hybrids and their evaluation

The selected BC_2_F_4_ (QβLM11, QβLM13 as a seed parent and QβLM12, QβLM14 as a male parent) progenies of the improved inbreds were crossed in different combinations to reconstitute hybrids (RBuland and RPMH1) during 2019 (*spring* season) at Punjab Agricultural University, Ludhiana. The nutritionally enriched reconstituted hybrids with their corresponding original hybrids were raised in two rows of 3 m row length with three replications in a randomized complete block design (RBCD) and were evaluated for their agronomic performance at three locations viz*.* (i) PAU, Ludhiana, (ii) Regional Research Station (PAU) Gurdaspur and (iii) IARI, New Delhi during 2019 (*rainy* season). To enhance the efficiency of selection various morphological characteristics were recorded as per DUS (Distinctness, Uniformity, and Stability) guidelines. Lysine, tryptophan, β-carotene, β-cryptoxanthin, and proA content were estimated from selfed seeds of reconstituted hybrid versions with the combination of three genes i.e. *crtRB1*, *lcyE*, and *o2*, along with their corresponding original hybrids. For each entry the grains were divided into three parts and analyzed as three replicates for estimation of different mineral nutrients viz*.*, Fe, Zn, Cu, Se, Mn, S, K, P, and Mg using simultaneous multi-element inductively coupled plasma–optical emission spectrometer (ICP-OES, Perkin Elmer) as per the methodology described by Arora et al.^57^. The value obtained was then multiplied by initial sample volume, divided by initial weight of grains, and expressed as μg element g^−1^ dry grain material (ppm)^58^.

### Assessment of reconstituted hybrids for *chapatti* (flatbread) making quality

Nutritionally enriched reconstituted hybrids with their corresponding checks along-with a local market sample were used for *chapatti* formulation. Whole grain flour was obtained by grinding the grains in domestic attachakki (burr mill) using the mesh sieves of 60 µm particle size. 100 gm of flour was mixed with optimum water to make dough and was then allowed to rest for 20–30 min. The optimum level of water for dough development was recorded in all the reconstituted hybrids. Dough ball of 60 g each was rolled and baked on a hot skillet; until brown spots appeared on it. The *chapattis* dough was evaluated for dough handling, roll-ability, and puffing. Further, the maize grains were subjected to a nixtamalization process^53^ to make soft dough known as “Massa”. This Massa was then used for the production of tortillas which are the main source of carbohydrates and calcium^54^. Sensory properties like appearance, color, flavor, texture, and overall acceptability were assessed using ten points Hedonic scale (9 for maximum and 0 for minimum) by the panel of sixteen semi-trained judges^59^. The panelists (8 males and 8 females) provided signed written informed consent for assessment of sensory parameters. This study was approved by the local ethics committee of Department of Food Science and Technology, PAU, Ludhiana. All methods were carried out in accordance with relevant guidelines and regulations.

## Supplementary Information

Supplementary Information.
