# Supplementary material for: Marker-assisted pyramiding of lycopene-ε-cyclase, β-carotene hydroxylase1 and opaque2 genes for development of biofortified maize hybrids
Source: Sci Rep. 2021 Jun 16;11:12642. doi: 10.1038/s41598-021-92010-8 (PMC8209105; doi:10.1038/s41598-021-92010-8)
Supplement: Supplementary file 1 — Supplementary Information. [file 41598_2021_92010_MOESM1_ESM.docx]

**Marker-assisted pyramiding of *lycopene-*ε*-cyclase,* *β-carotene hydroxylase1* and *opaque2* genes for development of biofortified maize hybrids**

Jagveer Singh^1^, Shikha Sharma^1^, Amandeep Kaur^1^, Yogesh Vikal^1*^, Amandeep Kaur Cheema^1^, Balraj Kaur Bains^1^, Noorpreet Kaur^2^, Gurjit Kaur Gill^2^, Pawan Kumar Malhotra^1^, Ashok Kumar^3^, Priti Sharma^1^, Vignesh Muthusamy^5^, Amarjeet Kaur^4^, Jasbir Singh Chawla^2^ and Firoz Hossain^5^

^1^School of Agricultural Biotechnology, Punjab Agricultural University, Ludhiana, India

^2^Department of Plant Breeding and Genetics, Punjab Agricultural University, Ludhiana, India

^3^Department of Plant Breeding and Genetics, Regional Research Station, Gurdaspur, India

^4^Department of Plant Breeding and Genetics, Punjab Agricultural University, Ludhiana, India

^5^Division of Genetics, Indian Agricultural Research Institute, New Delhi, India

***Corresponding author:*** (Email id: [yvikal-soab@pau.edu](mailto:yvikal-soab@pau.edu))

**Supplementary Table S1.** List of SSR markers analysed for parental polymorphism and percentage polymorphism observed in four crosses.

| **Chromosome No.** | **No. of markers analysed** | **Polymorphic markers among respective recurrent parent and donor parent** | | | | | | | |
| --- | --- | --- | --- | --- | --- | --- | --- | --- | --- |
|  |  | **QLM11 × HP-467-15** | | **QLM12 × HP-467-15** | | **QLM13 × HP-467-15** | | **QLM14 × HP-467-15** | |
|  |  | **NP** | **Pol. (%)** | **NP** | **Pol. (%)** | **NP** | **Pol. (%)** | **NP** | **Pol. (%)** |
| Chromosome 1 | 40.0 | 18.0 | 45.0 | 12.0 | 30.0 | 18.0 | 45.0 | 11.0 | 27.5 |
| Chromosome 2 | 48.0 | 26.0 | 52.0 | 27.0 | 54.0 | 18.0 | 36.0 | 11.0 | 22.0 |
| Chromosome 3 | 55.0 | 16.0 | 28.5 | 21.0 | 37.5 | 23.0 | 41.0 | 11.0 | 19.0 |
| Chromosome 4 | 44.0 | 17.0 | 37.7 | 17.0 | 37.0 | 14.0 | 31.0 | 11.0 | 24.0 |
| Chromosome 5 | 20.0 | 12.0 | 60.0 | 12.0 | 60.0 | 6.0 | 30.0 | 8.0 | 40.0 |
| Chromosome 6 | 25.0 | 16.0 | 64.0 | 7.0 | 28.0 | 18.0 | 72.0 | 17.0 | 68.0 |
| Chromosome 7 | 31.0 | 18.0 | 62.0 | 14.0 | 48.0 | 18.0 | 62.0 | 21.0 | 72.4 |
| Chromosome 8 | 14.0 | 7.0 | 50.0 | 7.0 | 50.0 | 8.0 | 57.0 | 8.0 | 57.0 |
| Chromosome 9 | 27.0 | 16.0 | 64.0 | 9.0 | 36.0 | 11.0 | 44.0 | 12.0 | 48.0 |
| Chromosome 10 | 20.0 | 15.0 | 75.0 | 8.0 | 40.0 | 10.0 | 50.0 | 10.0 | 50.0 |
| Total | 324 | 161.0 | - | 134.0 | - | 144.0 | - | 120.0 | - |
| % of polymorphism | - | 49.69% | - | 41.35% | - | 44.44% | - | 37.03% | - |

NP, number of observed polymorphic markers; Pol. (%), polymorphism percentage.

**Supplementary Table S2.** Distinguish morphological qualitative traits of QPM recurrent parents used in present study.

| **Recurrent parent** | **Leaf attitude** | **Anther glume color** | **Tassel density** | **Tassel shape** | **Anther color** | **Silk color** | **Ear shape** | **Kernel color** |
| --- | --- | --- | --- | --- | --- | --- | --- | --- |
| QLM11 | Semi-drooping | Green | Lax | Strongly curved | Pink | Pink | Cylindrical | Orange red |
| QLM12 | Drooping | Green | Medium | Straight | Cream | Cream | Conical | Dull orange |
| QLM13 | Semi-errect | Pink | Dense | Straight | Pink | Pink | Conico Cylindrical | Yellow |
| QLM14 | Semi-erect | Green | Medium | Curved | Creamish pink | Green | Conico Cylindrical | Yellow |

**Supplementary Table S3.** Agronomic Performance of selected introgressed progenies *vis-à-vis* respective recurrent parent at three locations.

| **Introgressed**  **Inbreds** | **DTA** | | | **DTS** | | | **PH (cm)** | | | **EH (cm)** | | |
| --- | --- | --- | --- | --- | --- | --- | --- | --- | --- | --- | --- | --- |
|  | **LDH** | **GSP** | **DEL** | **LDH** | **GSP** | **DEL** | **LDH** | **GSP** | **DEL** | **LDH** | **GSP** | **DEL** |
| QβLM11-A | 55.0 | 53.3 | 53.0 | 57.7 | 56.0 | 56.3 | 159.2 | 181.0 | 152.2 | 94.6 | 100.5 | 75.0 |
| QβLM11-B | 55.5 | 54.0 | 50.3 | 59.0 | 56.0 | 54.3 | 162.0 | 180.7 | 153.3 | 96.0 | 100.3 | 79.8 |
| QβLM11-C | 56.0 | 55.3 | 51.3 | 59.3 | 58.0 | 53.3 | 167.6 | 175.1 | 143.0 | 98.8 | 97.5 | 74.7 |
| QLM11 | 53.0 | 54.0 | 52.3 | 55.3 | 56.3 | 55.0 | 155.6 | 191.8 | 150.8 | 92.8 | 105.9 | 78.8 |
| QβLM12-A | 54.3 | 53.3 | 53.0 | 57.0 | 57.3 | 56.0 | 159.0 | 166.6 | 134.8 | 94.5 | 93.3 | 70.5 |
| QβLM12-B | 55.3 | 53.3 | 54.0 | 57.3 | 56.0 | 56.3 | 164.8 | 170.6 | 146.7 | 97.4 | 95.3 | 77.2 |
| QβLM12-C | 54.0 | 52.3 | 52.0 | 56.3 | 54.3 | 55.3 | 176.6 | 192.5 | 132.3 | 103.3 | 106.3 | 75.3 |
| QLM12 | 52.3 | 53.0 | 52.3 | 56.3 | 56.0 | 55.0 | 176.7 | 195.0 | 147.8 | 103.3 | 107.5 | 80.7 |
| QβLM13-A | 55.3 | 53.3 | 53.0 | 58.0 | 57.3 | 56.0 | 168.1 | 167.5 | 157.7 | 99.1 | 93.8 | 82.7 |
| QβLM13-B | 56.3 | 53.0 | 52.3 | 58.3 | 56.0 | 54.3 | 164.8 | 187.5 | 178.0 | 97.4 | 103.8 | 98.3 |
| QβLM13-C | 57.0 | 53.3 | 51.3 | 60.0 | 55.3 | 55.3 | 164.6 | 157.5 | 162.3 | 97.3 | 88.8 | 94.3 |
| QLM13 | 55.3 | 52.3 | 54.0 | 58.0 | 54.3 | 56.3 | 150.4 | 175.0 | 168.3 | 90.2 | 97.5 | 93.5 |
| QβLM14-A | 58.3 | 52.3 | 55.0 | 61.0 | 55.0 | 58.0 | 170.8 | 190.0 | 138.3 | 100.4 | 105.0 | 79.3 |
| QβLM14-B | 56.3 | 54.0 | 55.3 | 59.0 | 57.0 | 58.3 | 161.0 | 185.0 | 146.8 | 95.5 | 102.5 | 88.8 |
| QβLM14-C | 56.3 | 53.3 | 55.0 | 59.3 | 56.3 | 57.3 | 160.8 | 185.0 | 153.5 | 95.4 | 102.5 | 89.2 |
| QLM14 | 58.3 | 53.0 | 54.3 | 61.0 | 55.0 | 56.3 | 182.6 | 182.5 | 151.8 | 106.3 | 101.3 | 87.2 |
| SE(d) | 1.2 | 1.3 | 0.5 | 1.1 | 1.2 | 0.4 | 3.7 | 3.8 | 2.6 | 1.8 | 2.1 | 1.9 |
| CD (0.05%) | 3.2 | 3.6 | 1.4 | 3.2 | 3.3 | 1.2 | 10.3 | 10.6 | 7.2 | 4.9 | 5.8 | 5.4 |

DTA, days to 50% anthesis; DTS, days to 50% silking; PH, plant height; EH, ear height; LDH, Ludhiana; GSP, Gurdaspur; DEL, Delhi; SE, standard error of difference; CD, critical difference for comparing the averages among sets.

**Supplementary Table S4.** Agronomic performance of selected introgressed progenies *vis-à-vis* respective recurrent parent at three locations.

| **Introgressed**  **inbreds** | **CG (cm)** | | | **CL (cm)** | | | **NRPE** | | | **NKPR** | | | **GY (kg/ha)** | | |
| --- | --- | --- | --- | --- | --- | --- | --- | --- | --- | --- | --- | --- | --- | --- | --- |
|  | **LDH** | **GSP** | **DEL** | **LDH** | **GSP** | **DEL** | **LDH** | **GSP** | **DEL** | **LDH** | **GSP** | **DEL** | **LDH** | **GSP** | **DEL** |
| QβLM11-A | 3.8 | 4.0 | 3.3 | 16.6 | 16.7 | 16.2 | 15.6 | 16.8 | 14.3 | 25.9 | 26.1 | 21.0 | 3538.9 | 3740.9 | 3253.3 |
| QβLM11-B | 3.3 | 3.4 | 2.5 | 15.3 | 14.5 | 13.8 | 15.4 | 14.7 | 12.3 | 20.4 | 18.3 | 16.5 | 3274.7 | 3209.0 | 3022.2 |
| QβLM11-C | 3.8 | 3.5 | 3.2 | 14.1 | 17.3 | 17.0 | 19.7 | 18.3 | 14.7 | 25.6 | 26.6 | 21.8 | 3347.0 | 3175.4 | 2542.2 |
| QLM11 | 3.3 | 3.8 | 3.0 | 15.5 | 16.7 | 14.6 | 16.5 | 18.7 | 13.0 | 20.0 | 21.6 | 17.8 | 2999.7 | 3558.3 | 2844.4 |
| QβLM12-A | 3.8 | 4.1 | 3.3 | 16.8 | 15.3 | 11.9 | 16.3 | 20.5 | 12.3 | 22.0 | 20.8 | 17.0 | 3451.0 | 3890.2 | 3164.4 |
| QβLM12-B | 3.4 | 3.9 | 3.0 | 17.0 | 20.0 | 12.3 | 17.7 | 19.0 | 12.0 | 22.7 | 25.3 | 18.2 | 3375.0 | 3586.9 | 3288.9 |
| QβLM12-C | 3.4 | 3.3 | 2.9 | 18.9 | 15.0 | 11.0 | 14.0 | 14.7 | 12.0 | 27.3 | 20.3 | 16.5 | 3012.0 | 3070.7 | 2968.9 |
| QLM12 | 3.6 | 4.0 | 3.3 | 15.8 | 17.0 | 12.1 | 14.7 | 15.0 | 13.3 | 20.3 | 23.0 | 17.5 | 3080.6 | 3597.7 | 3057.8 |
| QβLM13-A | 4.0 | 3.9 | 2.9 | 17.5 | 17.5 | 13.4 | 17.7 | 17.0 | 11.3 | 27.6 | 28.3 | 21.3 | 3532.9 | 3345.5 | 3395.6 |
| QβLM13-B | 4.2 | 4.3 | 3.5 | 16.8 | 18.2 | 17.1 | 16.3 | 16.7 | 12.3 | 25.4 | 27.7 | 25.0 | 3546.4 | 3735.0 | 3573.3 |
| QβLM13-C | 3.4 | 4.1 | 3.9 | 15.9 | 18.2 | 14.7 | 12.1 | 12.0 | 11.3 | 26.7 | 25.9 | 25.0 | 3359.3 | 3571.8 | 3075.6 |
| QLM13 | 3.8 | 4.1 | 3.3 | 16.6 | 17.5 | 14.3 | 15.7 | 17.3 | 12.3 | 24.3 | 25.5 | 23.2 | 3074.2 | 3370.0 | 3146.7 |
| QβLM14-A | 3.8 | 3.6 | 3.0 | 15.2 | 17.3 | 12.8 | 15.8 | 14.8 | 14.3 | 20.7 | 21.4 | 17.2 | 3372.1 | 3557.1 | 3040.0 |
| QβLM14-B | 4.0 | 3.5 | 3.3 | 18.1 | 18.3 | 16.8 | 16.7 | 17.3 | 15.3 | 23.2 | 22.3 | 20.8 | 3648.8 | 3225.5 | 3395.6 |
| QβLM14-C | 3.2 | 3.1 | 2.9 | 16.2 | 18.0 | 15.5 | 16.4 | 16.9 | 15.0 | 20.1 | 22.3 | 19.0 | 3265.8 | 3546.4 | 3466.7 |
| QLM14 | 3.2 | 3.5 | 3.1 | 18.5 | 14.2 | 15.7 | 17.2 | 16.4 | 16.0 | 22.2 | 18.7 | 19.3 | 3425.3 | 3446.4 | 3253.3 |
| SE(d) | 0.1 | 0.1 | 0.1 | 0.5 | 0.4 | 0.4 | 0.5 | 0.5 | 0.2 | 0.8 | 0.7 | 1.0 | 126.5 | 125.4 | 80.6 |
| CD (0.05%) | 0.3 | 0.3 | 0.2 | 1.3 | 1.1 | 1.1 | 1.3 | 1.3 | 0.7 | 2.3 | 1.9 | 2.8 | 350.6 | 347.6 | 223.3 |

CG, cob girth; CL, cob length; NRPE, number of rows per ear; NKPR, number of kernels per row; GY, grain yield; LDH, Ludhiana; GSP, Gurdaspur; DEL, Delhi; SE, standard error of difference; CD, critical difference for comparing the averages among sets.

**Supplementary Table S5.** Agronomic performance of selected reconstituted hybrids *vis-à-vis* original hybrids at three locations.

| **Reconstituted**  **hybrids** | **DTA** | | | **DTS** | | | **PH (cm)** | | | **EH (cm)** | | |
| --- | --- | --- | --- | --- | --- | --- | --- | --- | --- | --- | --- | --- |
|  | **LDH** | **GSP** | **DEL** | **LDH** | **GSP** | **DEL** | **LDH** | **GSP** | **DEL** | **LDH** | **GSP** | **DEL** |
| RBuland-2 | 52.3 | 55.3 | 52.3 | 55.3 | 58.0 | 54.0 | 189.7 | 175.1 | 183.0 | 109.9 | 97.5 | 111.8 |
| RBuland-4 | 53.3 | 53.3 | 53.3 | 56.0 | 55.3 | 56.3 | 187.6 | 178.8 | 195.8 | 108.8 | 99.4 | 99.5 |
| RBuland-6 | 54.0 | 55.0 | 52.0 | 57.0 | 58.0 | 55.3 | 184.1 | 193.6 | 192.5 | 107.0 | 106.8 | 106.5 |
| RBuland-8 | 51.3 | 56.0 | 54.0 | 55.0 | 58.0 | 57.0 | 151.4 | 215.0 | 201.3 | 90.7 | 117.5 | 100.8 |
| RBuland-11 | 53.0 | 56.0 | 53.0 | 55.0 | 58.0 | 55.3 | 187.4 | 157.5 | 208.5 | 108.7 | 88.8 | 113.0 |
| Buland | 53.0 | 53.0 | 52.0 | 55.7 | 56.3 | 56.0 | 156.1 | 187.5 | 205.7 | 93.1 | 103.8 | 112.2 |
| RPMH1-2 | 56.3 | 54.0 | 52.3 | 58.3 | 56.3 | 55.3 | 163.1 | 205.0 | 186.0 | 96.5 | 112.5 | 119.3 |
| RPMH1-17 | 56.0 | 52.3 | 54.3 | 57.7 | 54.3 | 58.0 | 184.4 | 175.0 | 203.5 | 107.2 | 97.5 | 109.2 |
| RPMH1-24 | 58.3 | 55.3 | 55.3 | 60.3 | 58.0 | 57.3 | 180.1 | 205.0 | 196.5 | 105.0 | 112.5 | 112.0 |
| RPMH1-4 | 57.0 | 53.0 | 52.0 | 59.7 | 56.3 | 55.0 | 182.8 | 193.0 | 214.5 | 106.4 | 106.5 | 118.8 |
| RPMH1-8 | 53.3 | 53.3 | 53.3 | 57.0 | 56.0 | 56.3 | 192.4 | 167.5 | 190.3 | 111.2 | 93.8 | 101.3 |
| PMH1 | 56.0 | 55.3 | 54.0 | 60.0 | 58.3 | 56.3 | 192.4 | 182.5 | 213.3 | 111.2 | 101.3 | 117.8 |
| HQPM1 | 57.3 | 53.0 | 50.3 | 59.7 | 56.0 | 53.0 | 183.1 | 187.5 | 207.0 | 106.6 | 103.8 | 116.8 |
| SE(m)+ | 1.4 | 1.5 | 0.5 | 1.4 | 1.1 | 0.5 | 3.1 | 3.5 | 3.0 | 1.6 | 2.3 | 2.9 |
| CD (0.05%) | 3.8 | 4.0 | 1.4 | 3.8 | 3.2 | 1.4 | 8.6 | 9.8 | 8.2 | 4.4 | 6.5 | 7.9 |

DTA, days to 50% anthesis; DTS, days to 50% silking; PH, plant height; EH, ear height; LDH, Ludhiana; GSP, Gurdaspur; DEL, Delhi; SE, standard error of difference; CD critical difference for comparing the averages among sets.

**Supplementary Table S6.** Agronomic performance of selected reconstituted hybrids *vis-à-vis* original hybrids at three locations.

| **Reconstituted**  **hybrids** | **CG (cm)** | | | **CL (cm)** | | | **NRPE** | | | **NKPR** | | | **GY (kg/ha)** | | |
| --- | --- | --- | --- | --- | --- | --- | --- | --- | --- | --- | --- | --- | --- | --- | --- |
|  | **LDH** | **GSP** | **DEL** | **LDH** | **GSP** | **DEL** | **LDH** | **GSP** | **DEL** | **LDH** | **GSP** | **DEL** | **LDH** | **GSP** | **DEL** |
| RBuland-2 | 4.9 | 5.2 | 4.4 | 17.1 | 17.8 | 17.8 | 16.0 | 16.3 | 15.7 | 30.3 | 32.2 | 31.5 | 6730.4 | 6926.7 | 6702.2 |
| RBuland-4 | 4.6 | 4.9 | 4.5 | 18.9 | 19.3 | 18.4 | 14.8 | 15.7 | 14.3 | 32.5 | 33.7 | 32.2 | 7185.2 | 7632.7 | 7022.2 |
| RBuland-6 | 4.5 | 4.3 | 4.4 | 15.6 | 17.3 | 17.2 | 15.1 | 14.9 | 14.7 | 28.7 | 30.0 | 29.5 | 7265.6 | 6912.6 | 6577.8 |
| RBuland-8 | 3.9 | 4.3 | 4.6 | 15.0 | 15.7 | 16.7 | 13.8 | 14.7 | 14.0 | 34.8 | 35.4 | 34.3 | 6856.7 | 7352.6 | 6755.6 |
| RBuland-11 | 4.9 | 4.4 | 4.9 | 19.6 | 21.7 | 20.4 | 17.5 | 16.7 | 15.3 | 25.6 | 37.0 | 35.8 | 7375.6 | 7345.0 | 7217.8 |
| Buland | 5.7 | 4.8 | 4.8 | 18.1 | 20.3 | 19.3 | 18.5 | 15.0 | 15.7 | 32.2 | 36.4 | 34.7 | 6739.0 | 7229.3 | 6791.1 |
| RPMH1-2 | 4.5 | 5.1 | 4.8 | 20.6 | 21.8 | 20.0 | 15.7 | 15.3 | 13.0 | 36.0 | 36.3 | 35.5 | 8432.2 | 8966.3 | 8320.0 |
| RPMH1-17 | 4.6 | 4.9 | 5.1 | 17.0 | 20.8 | 20.6 | 15.2 | 14.6 | 13.7 | 30.6 | 33.6 | 32.2 | 8353.1 | 8249.9 | 8266.7 |
| RPMH1-24 | 5.3 | 5.0 | 4.9 | 22.0 | 19.7 | 20.3 | 19.5 | 17.8 | 13.7 | 37.4 | 32.0 | 35.7 | 8270.2 | 7293.5 | 7644.4 |
| RPMH1-4 | 5.2 | 5.3 | 5.2 | 23.7 | 23.5 | 22.1 | 18.5 | 18.9 | 14.7 | 37.6 | 36.7 | 36.8 | 8481.4 | 8595.3 | 8231.1 |
| RPMH1-8 | 4.1 | 4.3 | 4.9 | 16.0 | 18.9 | 17.5 | 12.3 | 11.3 | 12.7 | 28.7 | 32.6 | 30.7 | 7863.7 | 7654.7 | 8337.8 |
| PMH1 | 4.8 | 5.7 | 5.4 | 18.6 | 21.7 | 21.3 | 13.0 | 15.2 | 13.7 | 34.6 | 37.5 | 35.8 | 7642.8 | 8376.0 | 7928.9 |
| HQPM1 | 5.1 | 4.1 | 5.2 | 17.7 | 19.8 | 18.8 | 14.7 | 13.5 | 13.7 | 33.5 | 34.8 | 37.8 | 6610.8 | 6590.6 | 7360.0 |
| SE(d) | 0.1 | 0.1 | 0.1 | 0.5 | 0.4 | 0.3 | 0.6 | 0.4 | 0.3 | 0.9 | 0.7 | 0.6 | 154.4 | 153.5 | 341.5 |
| CD (0.05%) | 0.3 | 0.2 | 0.3 | 1.4 | 1.2 | 0.9 | 1.6 | 1.1 | 0.9 | 2.4 | 2.0 | 1.7 | 428.0 | 425.5 | 946.4 |

CG, cob girth; CL, cob length; NRPE, number of rows per ear; NKPR, number of kernels per row; GY, grain yield; LDH, Ludhiana; GSP, Gurdaspur; DEL, Delhi; SE, standard error of difference; CD, critical difference for comparing the averages among sets.

**Supplementary Table S7.** Biochemical evaluation of selected reconstituted hybrid versions with respect to their original hybrids.

| **Reconstituted hybrids** | **Lysine (%)** | **Tryptophan (%)** | **BC (ppm)** | **BCX (ppm)** | **proA (ppm)** |
| --- | --- | --- | --- | --- | --- |
| RBULAND-2 | 0.313 | 0.074 | 6.48 | 3.52 | 8.24 |
| RBULAND-4 | 0.404 | 0.085 | 7.02 | 5.59 | 9.82 |
| RBULAND-6 | 0.287 | 0.07 | 6.13 | 1.68 | 6.97 |
| RBULAND-8 | 0.333 | 0.079 | 5.51 | 3.41 | 7.22 |
| RBULAND-11 | 0.431 | 0.071 | 5.7 | 3.81 | 7.61 |
| BULAND | 0.171 | 0.03 | 1.03 | 0.66 | 1.36 |
| RPMH1-2 | 0.379 | 0.071 | 6.73 | 3.59 | 8.53 |
| RPMH1-17 | 0.324 | 0.079 | 5.76 | 3.8 | 7.66 |
| RPMH1-24 | 0.429 | 0.088 | 5.58 | 3.39 | 7.28 |
| RPMH1-4 | 0.335 | 0.09 | 6.68 | 5.4 | 9.39 |
| RPMH1-8 | 0.296 | 0.087 | 6.08 | 5.36 | 8.76 |
| PMH1 | 0.232 | 0.046 | 0.8 | 0.82 | 1.21 |
| HQPM1 | 0.357 | 0.084 | 1.66 | 0.8 | 2.06 |
| SE(d) | 0.02 | 0.0 | 0.27 | 0.26 | 0.28 |
| CD (0.05%) | 0.06 | 0.01 | 0.74 | 0.72 | 0.78 |

BC, Beta-carotene; BCX, Beta-cryptoxanthin; proA, Provitamin-A; SE, standard error of difference; CD, critical difference for comparing the averages among sets.

**Supplementary Table S8.** Analysis of micronutrients concentration for reconstituted maize hybrids *vis-à-vis* original hybrids.

| **Reconstituted hybrids** | **Zn(ppm)** | **Fe(ppm)** | **Cu(ppm)** | **Mn(ppm)** | **Se(ppm)** | **P(ppm)** | **K(ppm)** | **Mg(ppm)** | **S(ppm)** |
| --- | --- | --- | --- | --- | --- | --- | --- | --- | --- |
| RBuland-2 | 36.53 | 32.82 | 1.39 | 6.78 | 6.14 | 3596.93 | 11625.74 | 1386.04 | 2449.82 |
| RBuland-4 | 35.59 | 35.94 | 1.36 | 6.34 | 6.50 | 3546.14 | 12076.29 | 1324.80 | 2220.10 |
| RBuland-6 | 35.11 | 32.13 | 1.37 | 6.54 | 6.67 | 3351.59 | 11519.43 | 1331.01 | 2351.10 |
| RBuland-8 | 34.52 | 34.86 | 1.38 | 6.35 | 6.23 | 3532.79 | 11232.74 | 1228.75 | 2221.30 |
| RBuland-11 | 35.49 | 34.44 | 1.33 | 6.59 | 6.30 | 3009.41 | 11715.99 | 1373.46 | 2462.80 |
| Buland | 34.82 | 32.55 | 1.37 | 6.82 | 6.00 | 3507.94 | 10828.99 | 1394.03 | 2466.87 |
| RPMH1-2 | 49.34 | 35.39 | 1.46 | 7.95 | 6.43 | 3631.62 | 11563.34 | 1382.61 | 2498.52 |
| RPMH1-17 | 47.87 | 37.44 | 1.46 | 7.57 | 6.42 | 3769.5 | 11750.21 | 1343.35 | 2226.60 |
| RPMH1-24 | 49.32 | 38.75 | 1.45 | 6.81 | 5.97 | 3846.15 | 11775.54 | 1410.8 | 2221.40 |
| RPMH1-4 | 49.41 | 35.27 | 1.49 | 7.51 | 6.42 | 3736.17 | 11665.42 | 1318.43 | 2478.26 |
| RPMH1-8 | 48.08 | 35.87 | 1.45 | 6.53 | 6.51 | 3941.81 | 11158.68 | 1407.35 | 2473.37 |
| PMH1 | 48.3 | 37.1 | 1.43 | 7.39 | 6.29 | 3692.50 | 11515.00 | 1489.30 | 2363.80 |
| SE (d) | 0.35 | 0.53 | 0.04 | 0.16 | 0.14 | 0.60 | 0.83 | 0.52 | 10.42 |
| CD (0.05%) | 0.97 | 1.46 | 0.12 | 0.45 | 0.39 | 1.67 | 2.29 | 1.44 | 28.86 |

Zn, Zinc; Fe, Iron Cu,Copper Mn, Manganese Se, Selenium P, Phosphorus K, Potassium Mg, Magnesium S, Sulfur. SE, Standard Error of difference. The critical difference (CD) are for comparing the averages among sets.

**Supplementary Table S9.** Details of genetic material used in MABB.

| **Inbreds** | **Pedigree** | **Tryptophan %** |
| --- | --- | --- |
| QLM11 | LM11/CML170//2*LM11- 1020-2-4-201-------- | 0.94 |
| QLM12 | LM12/DMR7//2*LM12 1156-3 | 0.95 |
| QLM13 | LM13/CML165//2*LM13 1086 | 0.89 |
| QLM14 | LM14/CML162//2*LM14 1066-10-88-42-4 | 1.03 |

**Supplementary Table S10.** Details of populations generated under MABB.

| **S. No.** | **Generations** | **Seasons** | **Experimental location** |
| --- | --- | --- | --- |
| 1. | Crosses between recurrent parent and donor parent | *rainy* 2015 | IARI Experimental Farm, New Delhi |
| 2. | F_1_ | *spring* 2016 | SOAB, PAU, Ludhiana |
| 3. | BC_1_F_1_ | *rainy 2016* | SOAB, PAU, Ludhiana |
| 4. | BC_2_F_1_ | *spring* 2017 | SOAB, PAU, Ludhiana |
| 5. | BC_2_F_2_ | *rainy* 2017 | SOAB, PAU, Ludhiana |
| 6. | BC_2_F_3_ | *rainy* 2018 | SOAB, PAU, Ludhiana, RRS, Gurdaspur, PAU & IARI, New Delhi |
| 7. | BC_2_F_4_ and reconstitution of hybrids in original combination | *spring* 2018 | SOAB, PAU, Ludhiana |
| 8. | Evaluation of reconstituted hybrids | *rainy* 2019 | SOAB, PAU, Ludhiana, RRS, Gurdaspur, PAU & IARI, New Delhi |

**Supplementary Table S11.** Detailed list of SSR markers analysed for background selection.

| **S. No.** | **Primer** | **Bin No.** | **Repeats** | **Forward base (5' to 3')** | **Reverse base (5' to 3')** |
| --- | --- | --- | --- | --- | --- |
| 1 | *umc1160* | 1.01 | CCA | [CGTTTGATATGATGTGGAGATTCG](https://www.maizegdb.org/data_center/primer?id=235205) | [AAGCTTGTGAATGTTCTGGATGTC](https://www.maizegdb.org/data_center/primer?id=235206) |
| 2 | *umc1547* | 1.01 | (TCT)5 | [AGTAAACGAGACAAGCATGGTTCC](https://www.maizegdb.org/data_center/primer?id=273062) | [CTCGGCGACTTGAAGAAGTAAAAA](https://www.maizegdb.org/data_center/primer?id=273063) |
| 3 | *bnlg1124* | 1.01 | AG(20) | [TCTTCATCTCTCTATCAAACTGACA](https://www.maizegdb.org/data_center/primer?id=171118) | [TGGCACATCCACAAGAACAT](https://www.maizegdb.org/data_center/primer?id=171119) |
| 4 | *bnlg1014* | 1.01 | AG(14) | [CACGCTGTTTCAGACAGGAA](https://www.maizegdb.org/data_center/primer?id=171036) | [CGCCTGTGATTGCACTACAC](https://www.maizegdb.org/data_center/primer?id=171037) |
| 5 | *bnlg1429* | 1.02 | AG(20) | [CTCCTCGCAAGGATCTTCAC](https://www.maizegdb.org/data_center/primer?id=171290) | [AGCACCGTTTCTCGTGAGAT](https://www.maizegdb.org/data_center/primer?id=171291) |
| 6 | *umc2225* | 1.02 | (AGAGAGAGAGAGAG)4 | [TCGGCTGACATAATAAAACCATAGC](https://www.maizegdb.org/data_center/primer?id=616083) | [ATGCGAATTTTACCGGGTTTTT](https://www.maizegdb.org/data_center/primer?id=616084) |
| 7 | *bnlg1614* | 1.02 | AG(15) | [CCAACCCACCCAGAGGAGA](https://www.maizegdb.org/data_center/primer?id=171380) | [AGCGGGCGAGATCTTCAT](https://www.maizegdb.org/data_center/primer?id=171381) |
| 8 | *bnlg1627* | 1.02 | AG(19 | [CGGACGGGGGTTATTAAAAT](https://www.maizegdb.org/data_center/primer?id=171388) | [TGTGTTCGCAGAATCTCTCG](https://www.maizegdb.org/data_center/primer?id=171389) |
| 9 | *bnlg176* | 1.03 | NA | [AGTTCACGTCCAGCTGAATGACAG](https://www.maizegdb.org/data_center/primer?id=114504) | [CGCGCATCGCATGCTTATCCTA](https://www.maizegdb.org/data_center/primer?id=114505) |
| 10 | *umc1701* | 1.03 | (AC)7 | [GCGGCAGTACAACACGTAACAATA](https://www.maizegdb.org/data_center/primer?id=292450) | [CGCCAATAAACTGGAGGATAAGAA](https://www.maizegdb.org/data_center/primer?id=292451) |
| 11 | *umc1917* | 1.04 | (CTG)6 | [ACTTCCACTTCACCAGCCTTTTC](https://www.maizegdb.org/data_center/primer?id=301792) | [GGAAAGAAGAGCCGCTTGGT](https://www.maizegdb.org/data_center/primer?id=301793) |
| 12 | *umc1144* | 1.04 | (CT)8 | [ATGGCCCACTCATCATATCTCTGT](https://www.maizegdb.org/data_center/primer?id=235157) | [TGTGTTGATTAGCAGCGGATAAAA](https://www.maizegdb.org/data_center/primer?id=235158) |
| 13 | *umc1703* | 1.05 | (CTTT)5 | [ATTTTCTTGCTCACGTTCACTTCC](https://www.maizegdb.org/data_center/primer?id=292456) | [TAACGGCAGCATTACATTTCTTGA](https://www.maizegdb.org/data_center/primer?id=292457) |
| 14 | *umc2025* | 1.05 | (AGCT)4 | [CGCCGTAGTATTTGGTAGCAGAAG](https://www.maizegdb.org/data_center/primer?id=309051) | [TCTACCGCTCCTTCGTCCAGTA](https://www.maizegdb.org/data_center/primer?id=309052) |
| 15 | *bnlg1811* | 1.06 | AG(16) | [ACACAAGCCGACCAAAAAAC](https://www.maizegdb.org/data_center/primer?id=171490) | [GTAGTAGGAACGGGCGATGA](https://www.maizegdb.org/data_center/primer?id=171491) |
| 16 | *bnlg1615* | 1.06 | AG(18) | [CAGAAGGGGAGGAGGGATAC](https://www.maizegdb.org/data_center/primer?id=171656) | [ATTATGCTCAAGCACAGGGC](https://www.maizegdb.org/data_center/primer?id=171657) |
| 17 | *umc1035* | 1.06 | (CT)19 | [CTGGCATGATCACGCTATGTATG](https://www.maizegdb.org/data_center/primer?id=174751) | [TAACATCAGCAGGTTTGCTCATTC](https://www.maizegdb.org/data_center/primer?id=174752) |
| 18 | *bnlg1908* | 1.06 | AG(19) | [TCAGGCAGCAATGTTCAGAC](https://www.maizegdb.org/data_center/primer?id=171536) | [TGGAGTAGCTCACGTTGACG](https://www.maizegdb.org/data_center/primer?id=171537) |
| 19 | *umc1833* | 1.07 | (TG)8 | [TTACATGTACCCACATCCTTGCAG](https://www.maizegdb.org/data_center/primer?id=301540) | [CAGGGATCTGGGAGTATCCTCTTT](https://www.maizegdb.org/data_center/primer?id=301541) |
| 20 | *bnlg1556* | 1.07 | AG(18) | [ACCGACCTAAGCTATGGGCT](https://www.maizegdb.org/data_center/primer?id=171350) | [CCGGTTATAAACACAGCCGT](https://www.maizegdb.org/data_center/primer?id=171351) |
| 21 | *bnlg1025* | 1.07 | AG(23) | [TGGTGAAGGGGAAGATGAAG](https://www.maizegdb.org/data_center/primer?id=171050) | [CCGAGACGTGACTCCTAAGC](https://www.maizegdb.org/data_center/primer?id=171051) |
| 22 | *umc1706* | 1.07 | (TCG)5 | [ATCGAGAGGGGTAAATAAGGACGA](https://www.maizegdb.org/data_center/primer?id=292465) | [ACCAACCACGAGGCGATGTA](https://www.maizegdb.org/data_center/primer?id=292466) |
| 23 | *umc1099* | 1.07 | (CT)10TT(CT)8 | [TGTTGACATTGAGACAGAGTCACG](https://www.maizegdb.org/data_center/primer?id=194066) | [GTGCTCGTAGTAGGGGTTACAAGC](https://www.maizegdb.org/data_center/primer?id=194067) |
| 24 | *phi039* | 1.08 | AG | [ACCGTGTCTAATGTGTCCATACGG](https://www.maizegdb.org/data_center/primer?id=130821) | [CGTTAGGAGCTGGCTAGTCTCA](https://www.maizegdb.org/data_center/primer?id=130822) |
| 25 | *umc1928* | 1.08 | (AC)8 | [CACACTCACAGTTTCACACTGCAT](https://www.maizegdb.org/data_center/primer?id=301825) | [GAATCTCGTACAGTGGTGATGTGG](https://www.maizegdb.org/data_center/primer?id=301826) |
| 26 | *bnlg1643* | 1.08 | AG(24) | [ACCACCGTCCACCTCCAC](https://www.maizegdb.org/data_center/primer?id=171402) | [ATTGACCCCGTGACCCTC](https://www.maizegdb.org/data_center/primer?id=171403) |
| 27 | *umc1085* | 1.08 | (AG)17 | [TACTGTGATGTGGCGGTGCT](https://www.maizegdb.org/data_center/primer?id=194024) | [GCCACCTCTCACAGGTCTCAC](https://www.maizegdb.org/data_center/primer?id=194025) |
| 28 | *umc1383* | 1.08 | (GACG)6 | [CACACACATCGATCATGAGCATAC](https://www.maizegdb.org/data_center/primer?id=256259) | [GTGTACTACCATCAGACCCATCCA](https://www.maizegdb.org/data_center/primer?id=256260) |
| 29 | *umc1914* | 1.08 | (CAG)4 | [CAACATGAGCGTGCTAAATACTCG](https://www.maizegdb.org/data_center/primer?id=301783) | [ACAGGAACACATGAGGTCATCAAA](https://www.maizegdb.org/data_center/primer?id=301784) |
| 30 | *umc2047* | 1.09 | (GACT)4 | [GACAGACATTCCTCGCTACCTGAT](https://www.maizegdb.org/data_center/primer?id=309117) | [CTGCTAGCTACCAAACATTCCGAT](https://www.maizegdb.org/data_center/primer?id=309118) |
| 31 | *bnlg1597* | 1.09 | AG(34) | [GATAATCTCGTCTCGCCAGG](https://www.maizegdb.org/data_center/primer?id=171362) | [CATAAAAGGATGCCGACGAC](https://www.maizegdb.org/data_center/primer?id=171363) |
| 32 | *phi011* | 1.09 | AGC | [TGTTGCTCGGTCACCATACC](https://www.maizegdb.org/data_center/primer?id=111637) | [GCACACACACAGGACGACAGT](https://www.maizegdb.org/data_center/primer?id=111638) |
| 33 | *umc1082* | 1.09 | (AG)16 | [CCGACCATGCATAAGGTCTAGG](https://www.maizegdb.org/data_center/primer?id=194015) | [GCCTGCATAGAGAGGTGGTATGAT](https://www.maizegdb.org/data_center/primer?id=194016) |
| 34 | *phi055* | 1.09 | GAA | [GAGATCGTGTGCCCGCACC](https://www.maizegdb.org/data_center/primer?id=111677) | [TTCCTCCTGCTCCTCAGACGA](https://www.maizegdb.org/data_center/primer?id=111678) |
| 35 | *bnlg1671* | 1.10 | (AG)21 | [TCACGATCAGCAAGCAATTC](https://www.maizegdb.org/data_center/primer?id=171416) | [CCCCACCAACCTTAGAGTCA](https://www.maizegdb.org/data_center/primer?id=171417) |
| 36 | *umc1534* | 1.10 | (AAG)5 | [GACCCCAAATCTCTCCTTCCTC](https://www.maizegdb.org/data_center/primer?id=273023) | [TAGCTAAGCTTGTGCTTGCTCG](https://www.maizegdb.org/data_center/primer?id=273024) |
| 37 | *umc1331* | 1.11 | (GGT)10 | [TTATGAACGTGGTCGTGACTATGG](https://www.maizegdb.org/data_center/primer?id=248592) | [ATATCTGTCCCTCTCCCACCATC](https://www.maizegdb.org/data_center/primer?id=248593) |
| 38 | *phi064* | 1.11 | ATCC | [CCGAATTGAAATAGCTGCGAGAACCT](https://www.maizegdb.org/data_center/primer?id=111693) | [ACAATGAACGGTGGTTATCAACACGC](https://www.maizegdb.org/data_center/primer?id=111694) |
| 39 | *phi265454* | 1.11 | AGG | [CAAGCACCTCAACCTCTTCG](https://www.maizegdb.org/data_center/primer?id=256133) | [TCCACGCTGCTCACCTTC](https://www.maizegdb.org/data_center/primer?id=256134) |
| 40 | *umc2244* | 1.12 | (GGC)4 | [TGTGAGCCTGATTCCATTTTGAC](https://www.maizegdb.org/data_center/primer?id=616140) | [CATCGTGGGGACTAAAGCGAC](https://www.maizegdb.org/data_center/primer?id=616141) |
| 41 | *umc1622* | 2.00 | (AAG)5 | [CGCTACAAATCCTACTGGTGCTTT](https://www.maizegdb.org/data_center/primer?id=273287) | [CCTCGGATTTTCCAAAACATTTCT](https://www.maizegdb.org/data_center/primer?id=273288) |
| 42 | *umc1165* | 2.01 | (TA)6 | [TATCTTCAGACCCAAACATCGTCC](https://www.maizegdb.org/data_center/primer?id=235220) | [GTCGATTGATTTCCCGATGTTAAA](https://www.maizegdb.org/data_center/primer?id=235221) |
| 43 | *umc2245* | 2.01 | (CAA)7 | [GCCCTGTTATTGGAACAGTTTACG](https://www.maizegdb.org/data_center/primer?id=616143) | [CGTCGTCTTCGACATGTACTTCAC](https://www.maizegdb.org/data_center/primer?id=616144) |
| 44 | *bnlg1338* | 2.01 | AG(30) | [GTGCAGAATGCAGGCAATAG](https://www.maizegdb.org/data_center/primer?id=171254) | [GCAAATGTTTTCACACACACG](https://www.maizegdb.org/data_center/primer?id=171255) |
| 45 | *umc1542* | 2.02 | (AG)10 | [TAAAGCTATGATGGCACTTGCAGA](https://www.maizegdb.org/data_center/primer?id=273047) | [CATATTTGCCTTTGCCCTTTTGTA](https://www.maizegdb.org/data_center/primer?id=273048) |
| 46 | *bnlg2277* | 2.02 | AG(19) | [TTACGGTACCAATTCGCTCC](https://www.maizegdb.org/data_center/primer?id=171634) | [GACGACGCCATTTTCTGATT](https://www.maizegdb.org/data_center/primer?id=171635) |
| 47 | *umc1185* | 2.03 | (GC)8 | [AGTAAAAGAGGCAAGGACTACGGC](https://www.maizegdb.org/data_center/primer?id=235280) | [GCGGCGATATATACGAGGTTGT](https://www.maizegdb.org/data_center/primer?id=235281) |
| 48 | *bnlg2248* | 2.03 | AG(30) | [CCACCACATCCGTTACATCA](https://www.maizegdb.org/data_center/primer?id=171626) | [ACTTTGACACCGGCGAATAC](https://www.maizegdb.org/data_center/primer?id=171627) |
| 49 | *umc1845* | 2.03 | (AG)8 | [TGGTTGAACTGTTAAATCTGTCCTGA](https://www.maizegdb.org/data_center/primer?id=301576) | [TGGTAACCAGATTCCCACAGATG](https://www.maizegdb.org/data_center/primer?id=301577) |
| 50 | *bnlg1064* | 2.03 | AG(16) | [CTGGTCCGAGATGATGGC](https://www.maizegdb.org/data_center/primer?id=171082) | [TCCATTTCTGCATCTGCAAC](https://www.maizegdb.org/data_center/primer?id=171083) |
| 51 | *umc1580* | 2.04 | (CCG)5 | [GGTCTTCTTCCCCATGGACC](https://www.maizegdb.org/data_center/primer?id=273161) | [GGTGACGGTATCATACGTGGTGT](https://www.maizegdb.org/data_center/primer?id=273162) |
| 52 | *umc2249* | 2.04 | (GCCGGT)4 | [AGAAGGTCGTCGTCCTGGAAC](https://www.maizegdb.org/data_center/primer?id=616155) | [GCATAGACTCCCTGACAGCCAC](https://www.maizegdb.org/data_center/primer?id=616156) |
| 53 | *umc1259* | 2.04 | (GCG)4 | [CTCTTTGGTGGCAGAAGCAGAAT](https://www.maizegdb.org/data_center/primer?id=246208) | [TAGCTAAACTTGAGTGCTCTGCCC](https://www.maizegdb.org/data_center/primer?id=246209) |
| 54 | *umc1579* | 2.04 | (GCT)5 | [AAGATCAGCTAGCGAGAGAAGCAA](https://www.maizegdb.org/data_center/primer?id=273158) | [AGGAGGTCAGTGCTGCAGGT](https://www.maizegdb.org/data_center/primer?id=273159) |
| 55 | *umc1485* | 2.04 | (CTG)4 | [CAATACCATTACATACCCGCACAA](https://www.maizegdb.org/data_center/primer?id=256532) | [CCGGCCGTTTAATTTACTAGTGTG](https://www.maizegdb.org/data_center/primer?id=256533) |
| 56 | *bnlg108* | 2.04 | AG(11) | [AAAGATCATGGGCGTACCAG](https://www.maizegdb.org/data_center/primer?id=171096) | [CAGGAACCTGATGACCACCT](https://www.maizegdb.org/data_center/primer?id=171097) |
| 57 | *bnlg166* | 2.04 | AG(19) | [AACCAAGGTTCTTGGAGGCT](https://www.maizegdb.org/data_center/primer?id=171410) | [ACCATTGTATTTTCCTAGAGAATCG](https://www.maizegdb.org/data_center/primer?id=171411) |
| 58 | *umc1454* | 2.04 | (CAT)5 | [GAGTCTACAATTACCTGGCCGAGA](https://www.maizegdb.org/data_center/primer?id=256439) | [ATGTACCCCGCATTTGTGTACCT](https://www.maizegdb.org/data_center/primer?id=256440) |
| 59 | *umc1007* | 2.04 | (CT)12 | [AAGCAATATCACTACTTTCCAGCC](https://www.maizegdb.org/data_center/primer?id=167248) | [TACGTAATTCGTAGCCTTGGTCC](https://www.maizegdb.org/data_center/primer?id=167249) |
| 60 | *bnlg381* | 2.04 | NA | [TCCCTCTTGAGTGTTTATCACAAA](https://www.maizegdb.org/data_center/primer?id=114513) | [GTTTCCATGGGCAGGTGTAT](https://www.maizegdb.org/data_center/primer?id=114514) |
| 61 | *bnlg1018* | 2.04 | AG(16) | [CGAGGTTAGCACCGACAAAT](https://www.maizegdb.org/data_center/primer?id=171042) | [CGAGTAAATGCTCTGTGCCA](https://www.maizegdb.org/data_center/primer?id=171043) |
| 62 | *umc2248* | 2.04 | (TATATA)5 | [CTCCGGTTTAATTTCTCCTCGAC](https://www.maizegdb.org/data_center/primer?id=616152) | [GGAACCCATCTCGCTACTAGCTC](https://www.maizegdb.org/data_center/primer?id=616153) |
| 63 | *umc2030* | 2.04 | (CGA)4 | [CTTCAGCAACCGGAGACGAG](https://www.maizegdb.org/data_center/primer?id=309066) | [GATGCAGTGTGCCAATAAAGATGA](https://www.maizegdb.org/data_center/primer?id=309067) |
| 64 | *umc2032* | 2.04 | (CCT)5 | [TCTATCATTCGAGTCAAGAAGCCA](https://www.maizegdb.org/data_center/primer?id=309072) | [AAAAGAAGACGGATTTCTTCGGAC](https://www.maizegdb.org/data_center/primer?id=309073) |
| 65 | *umc1285* | 2.04 | (ACG)4 | [TAAATATACGGCCCCAAGAAAACC](https://www.maizegdb.org/data_center/primer?id=246286) | [AAACTGGATATGGTTGGTTGGTTG](https://www.maizegdb.org/data_center/primer?id=246287) |
| 66 | *umc2088* | 2.05 | (GGA)4 | [ACGACAAGAAGGAGGCCAAAG](https://www.maizegdb.org/data_center/primer?id=484858) | [CAAGTAGATCGATCGAGCAGCAG](https://www.maizegdb.org/data_center/primer?id=484859) |
| 67 | *umc1884* | 2.05 | (TC)8 | [TAGGAGCAGTATGAGAGGGCACTT](https://www.maizegdb.org/data_center/primer?id=301693) | [CTTTTTCTAGCGATCATTCTCCCA](https://www.maizegdb.org/data_center/primer?id=301694) |
| 68 | *umc1581* | 2.05 | (AT)6 | [GGCACGAGATTTGAAACAGTTAAGA](https://www.maizegdb.org/data_center/primer?id=273164) | [ACTCCATTACCTTTGTCTTGGCAG](https://www.maizegdb.org/data_center/primer?id=273165) |
| 69 | *umc1028* | 2.06 | (GA)18 | [CCCAGGTAAAATTCGCTAGCCT](https://www.maizegdb.org/data_center/primer?id=174597) | [GGAACAAGGAAAGCTGAATACACG](https://www.maizegdb.org/data_center/primer?id=174598) |
| 70 | *bnlg1887* | 2.06 | AG(20) | [CGAACCACTGTAGGCATGTG](https://www.maizegdb.org/data_center/primer?id=171526) | [ATCATGCAGAGCAGATGCAG](https://www.maizegdb.org/data_center/primer?id=171527) |
| 71 | *nc003* | 2.06 | AG | [ACCCTTGCCTTTACTGAAACACAACAGG](https://www.maizegdb.org/data_center/primer?id=130747) | [GCACACCGTGTGGCTGGTTC](https://www.maizegdb.org/data_center/primer?id=130748) |
| 72 | *umc2023* | 2.06 | (AGC)5 | [TCAGTCCCATTATATTCACCGACC](https://www.maizegdb.org/data_center/primer?id=309045) | [TCCTCTTCTTTTCCTCTCAGAGCC](https://www.maizegdb.org/data_center/primer?id=309046) |
| 73 | *umc1763* | 2.06 | (CT)8 | [TCTGCTGTCAAGGGATCGATTATT](https://www.maizegdb.org/data_center/primer?id=292636) | [GGCAAGAGGTTTCTCACTGATGAT](https://www.maizegdb.org/data_center/primer?id=292637) |
| 74 | *bnlg1225* | 2.06 | AG(14) | [GCAGTAGAAGAGCGAGCGAG](https://www.maizegdb.org/data_center/primer?id=171192) | [CATACGCTGTCACTGCCACT](https://www.maizegdb.org/data_center/primer?id=171193) |
| 75 | *bnlg1138* | 2.06 | AG(14) | [TGCTCTAGCCGACCTCAATT](https://www.maizegdb.org/data_center/primer?id=171130) | [ATGCCTGAACCGTGATTAGG](https://www.maizegdb.org/data_center/primer?id=171131) |
| 76 | *umc1080* | 2.06 | (GA)15 | [GAGGAGAAAAGGAGATGGAAAAGC](https://www.maizegdb.org/data_center/primer?id=194009) | [AGATGCCGCAGAAGATTCTAAACA](https://www.maizegdb.org/data_center/primer?id=194010) |
| 77 | *umc2253* | 2.06 | (TCGC)4 | [ACACCACGGGCTACTAGCTCAC](https://www.maizegdb.org/data_center/primer?id=616167) | [TCACGTCCAGGAGGATCACC](https://www.maizegdb.org/data_center/primer?id=616168) |
| 78 | *umc2178* | 2.06 | (TA)8 | [GTATCGAGACACGTACGCACGAA](https://www.maizegdb.org/data_center/primer?id=485128) | [GCGCAGTGATTTCTTACCATGC](https://www.maizegdb.org/data_center/primer?id=485129) |
| 79 | *umc1560* | 2.07 | (GC)6 | [CGTTCGTCTCTGGGTAGCGTAG](https://www.maizegdb.org/data_center/primer?id=273101) | [TATAACAGCCTGCTGCTGCTTG](https://www.maizegdb.org/data_center/primer?id=273102) |
| 80 | *phi090* | 2.08 | ATATC | [CTACCTATCCAAGCGATGGGGA](https://www.maizegdb.org/data_center/primer?id=130865) | [CGTGCAAATAATTCCCCGTGGGA](https://www.maizegdb.org/data_center/primer?id=130866) |
| 81 | *umc1464* | 2.08 | (CCA)6 | [CATGTAACTTGTGCCAAGAACACC](https://www.maizegdb.org/data_center/primer?id=256469) | [AAAGGAGGGTAGGAATAGCTGTGG](https://www.maizegdb.org/data_center/primer?id=256470) |
| 82 | *bnlg1141* | 2.08 | NA | [GAACTGGATTCCATCATCGG](https://www.maizegdb.org/data_center/primer?id=171136) | [AGGCTCAGCTGGCATTTAGA](https://www.maizegdb.org/data_center/primer?id=171137) |
| 83 | *umc1516* | 2.08 | (TA)12 | [CAATAACAGATCAGCATGGAGACG](https://www.maizegdb.org/data_center/primer?id=272969) | [TAGCGACCTGTTATCCGAAAGAAC](https://www.maizegdb.org/data_center/primer?id=272970) |
| 84 | *bnlg1606* | 2.08 | AG(23) | [TGTCCTTGTACCAGTGCTGC](https://www.maizegdb.org/data_center/primer?id=171374) | [GCTGTTCAGGATCTTCTGCC](https://www.maizegdb.org/data_center/primer?id=171375) |
| 85 | *bnlg1893* | 2.09 | AG(19) | [AATCCTGTAGCGTGTGTCCC](https://www.maizegdb.org/data_center/primer?id=171530) | [TAACTGAGTTGTTGAAGGAAATTG](https://www.maizegdb.org/data_center/primer?id=171531) |
| 86 | *umc1525* | 2.09 | (CGA)4 | [TTTGTGCCGAATATAAATGTGACG](https://www.maizegdb.org/data_center/primer?id=272996) | [AATAATATCAAATGGCGCCAAGC](https://www.maizegdb.org/data_center/primer?id=272997) |
| 87 | *umc2184* | 2.09 | (GCG)5 | [CTTGGCCTACTCCAAGTTCTCG](https://www.maizegdb.org/data_center/primer?id=485146) | [AGTAGAGCAGCACCATCCCG](https://www.maizegdb.org/data_center/primer?id=485147) |
| 88 | *umc1696* | 2.10 | (GA)8 | [CTAGGGTTTAACCAACGGGGAG](https://www.maizegdb.org/data_center/primer?id=292435) | [TAAGGAGAGGGTCGATGAACACAT](https://www.maizegdb.org/data_center/primer?id=292436) |
| 89 | *umc2118* | 3.00 | (CTTT)4 | [CGTCTCCGTCTGCAGTCACTATTA](https://www.maizegdb.org/data_center/primer?id=484948) | [TATGGTCCTCGGAGTTTGTTTGTT](https://www.maizegdb.org/data_center/primer?id=484949) |
| 90 | *umc1793* | 3.00 | (AT)6 | [TGCACACCCTTTATTGAATCATCA](https://www.maizegdb.org/data_center/primer?id=292726) | [CGTATAAGCTTTTTGGGGTCCTCT](https://www.maizegdb.org/data_center/primer?id=292727) |
| 91 | *phi453121* | 3.00 | ACC | [ACCTTGCCTGTCCTTCTTTCT](https://www.maizegdb.org/data_center/primer?id=256217) | [CAAGCAAGACTTTTGATCAGCC](https://www.maizegdb.org/data_center/primer?id=256218) |
| 92 | *phi104127* | 3.01 | ACCG | [CTTTGCTGCTGCTTCCTACG](https://www.maizegdb.org/data_center/primer?id=256094) | [AACCAGTGACGTACACAAAGCA](https://www.maizegdb.org/data_center/primer?id=256095) |
| 93 | *umc1970* | 3.01 | NA | [ACTGATGGTGTTCTTGGGTGTTTT](https://www.maizegdb.org/data_center/primer?id=301951) | [TTTTTACCCGAAGGTTCATCGTTT](https://www.maizegdb.org/data_center/primer?id=301952) |
| 94 | *umc1458* | 3.02 | (GCT)5 | [CCAATAAACAAATCATCTCCCCCT](https://www.maizegdb.org/data_center/primer?id=256451) | [TGCTATGCTATGTACAGGGACAGG](https://www.maizegdb.org/data_center/primer?id=256452) |
| 95 | *umc1886* | 3.02 | (CG)8 | [GTTTGACAGCACAAGTGCAAGAAA](https://www.maizegdb.org/data_center/primer?id=301699) | [GAGGTGGACATTGGACAACACC](https://www.maizegdb.org/data_center/primer?id=301700) |
| 96 | *bnlg1144* | 3.02 | NA | [TACTCGTCGTGTGGCGTTAG](https://www.maizegdb.org/data_center/primer?id=171138) | [AGCCGAGGCTATCTAACGGT](https://www.maizegdb.org/data_center/primer?id=171139) |
| 97 | *bnlg1325* | 3.03 | AG(18) | [CTAAATGCGCAGCAGTAGCA](https://www.maizegdb.org/data_center/primer?id=171242) | [TGCTCTGCAACAACTTGAGG](https://www.maizegdb.org/data_center/primer?id=171243) |
| 98 | *bnlg1447* | 3.03 | AG(33) | [GAGAGGAGAGGCTGAGCTGA](https://www.maizegdb.org/data_center/primer?id=171304) | [TCCTCCCACTGAATTTCCAC](https://www.maizegdb.org/data_center/primer?id=171305) |
| 99 | *bnlg1638* | 3.04 | AG(25) | [CATATCTCTAGCTTCTCGTCTTCG](https://www.maizegdb.org/data_center/primer?id=171396) | [ACACCGATCGAGGAAGAATG](https://www.maizegdb.org/data_center/primer?id=171397) |
| 100 | *umc2002* | 3.04 | NA | [TGACCTCAACTCAGAATGCTGTTG](https://www.maizegdb.org/data_center/primer?id=302047) | [CACAAAATCCTCGAGTTCTTGATTG](https://www.maizegdb.org/data_center/primer?id=302048) |
| 101 | *umc1386* | 3.04 | (CTCC)4 | [TACATTGCACGTTAGAAAACTGGC](https://www.maizegdb.org/data_center/primer?id=256268) | [GCTCACCAAGAGCTTCAAGGAG](https://www.maizegdb.org/data_center/primer?id=256269) |
| 102 | *umc1729* | 3.04 | (CGG)5 | [GTCGTACCACACCAGCCACA](https://www.maizegdb.org/data_center/primer?id=292534) | [TTCACTTCCACTTGTTGAACTTGC](https://www.maizegdb.org/data_center/primer?id=292535) |
| 103 | *umc1908* | 3.04 | (AAG)6 | [CGTACACTCAATCACGATCCAAAC](https://www.maizegdb.org/data_center/primer?id=301765) | [AACTTTGGGTACAAGTCAAGAGGC](https://www.maizegdb.org/data_center/primer?id=301766) |
| 104 | *umc1920* | 3.04 | (GT)8 | [GGTTCGGGTTTGCTACGTGTT](https://www.maizegdb.org/data_center/primer?id=301801) | [ACGAGACAACACAACCAAGACAAA](https://www.maizegdb.org/data_center/primer?id=301802) |
| 105 | *nc030* | 3.04 | CT | [CCCCTTGTCTTTCTTCCTCC](https://www.maizegdb.org/data_center/primer?id=111629) | [CGATTAGATTGGGGTGCG](https://www.maizegdb.org/data_center/primer?id=111630) |
| 106 | *umc1392* | 3.04 | (AGC)4 | [CTAAGATAGCAGCGACAACCGACT](https://www.maizegdb.org/data_center/primer?id=256286) | [GCCTGCTCTAAAGACATTCGTCC](https://www.maizegdb.org/data_center/primer?id=256287) |
| 107 | *umc1742* | 3.04 | (CGC)4 | [GCTTCCATCCTACTGATGATGGTT](https://www.maizegdb.org/data_center/primer?id=292573) | [AAGCTGATGCTTTGCCCTTG](https://www.maizegdb.org/data_center/primer?id=292574) |
| 108 | *umc1087* | 3.04 | (GA)17 | [AATCGTTTACACGAGAAGCAAAGC](https://www.maizegdb.org/data_center/primer?id=194030) | [ATTTCTAACTGGTCGCGCTGTTT](https://www.maizegdb.org/data_center/primer?id=194031) |
| 109 | *bnlg1628* | 3.04 | AG(16) | [GTAGGGTTCAAGGAGGCACA](https://www.maizegdb.org/data_center/primer?id=171390) | [CTCTCTGGTGAGCTGGCTTT](https://www.maizegdb.org/data_center/primer?id=171391) |
| 110 | *umc1683* | 3.04 | (CT)6 | [CGTCCGAAACTGTCTCTTCCTC](https://www.maizegdb.org/data_center/primer?id=292396) | [GCCTCTTCTATGGAGAGACTGGAG](https://www.maizegdb.org/data_center/primer?id=292397) |
| 111 | *umc2260* | 3.04 | (ATC)5 | [CATTTTTGTGCCTTCCAAAGAAAC](https://www.maizegdb.org/data_center/primer?id=616188) | [GTTTCAGGCTGACAAGGTCAAGA](https://www.maizegdb.org/data_center/primer?id=616189) |
| 112 | *umc1030* | 3.04 | (CT)21 | [TCCAGAGAATGAGATGACAAGACG](https://www.maizegdb.org/data_center/primer?id=174641) | [CAGAATAACAGGAGATGAGACGCA](https://www.maizegdb.org/data_center/primer?id=174642) |
| 113 | *umc2033* | 3.04 | (GCG)4 | [TCTAGATCCCTAGAGTAGCTGCGG](https://www.maizegdb.org/data_center/primer?id=309075) | [CACTCACGCAAATTAGCACAACTT](https://www.maizegdb.org/data_center/primer?id=309076) |
| 114 | *umc2264* | 3.04 | (ATG)4 | [AATTAGCCCTAAGAGAGCCCATTC](https://www.maizegdb.org/data_center/primer?id=616200) | [TAGACCTGCTCCATCATCGTTATC](https://www.maizegdb.org/data_center/primer?id=616201) |
| 115 | *umc1750* | 3.05 | (AGC)5 | [CCACAACTCGCTGCTGTCAATA](https://www.maizegdb.org/data_center/primer?id=292597) | [AGGAGGCTGCTGACCCTTCTACT](https://www.maizegdb.org/data_center/primer?id=292598) |
| 116 | *bnlg420* | 3.05 | NA | [CTTGCGCTCTCCTCCCCTT](https://www.maizegdb.org/data_center/primer?id=114585) | [GGCCAGCTCACTGCTCACT](https://www.maizegdb.org/data_center/primer?id=114586) |
| 117 | *umc1501* | 3.05 | (AAG)5 | [CCACATTTGGCTGAATTTGTTGTA](https://www.maizegdb.org/data_center/primer?id=272924) | [CTTGTTGGCTAGAAATTTGCCTTG](https://www.maizegdb.org/data_center/primer?id=272925) |
| 118 | *umc2155* | 3.05 | (AG)8 | [CATGTGGACACCATAGACCATGA](https://www.maizegdb.org/data_center/primer?id=485059) | [CCAGAGTTAATGTGACCTAGGCGT](https://www.maizegdb.org/data_center/primer?id=485060) |
| 119 | *bnlg1601* | 3.05 | AG(24) | [CAGACCAGAGACCATCTGCA](https://www.maizegdb.org/data_center/primer?id=171370) | [ATCGTGCGCTAGTCCAGAGT](https://www.maizegdb.org/data_center/primer?id=171371) |
| 120 | *umc1300* | 3.05 | (CAATC)5 | [TCTGAACGCACTGGGAACATAGTA](https://www.maizegdb.org/data_center/primer?id=246331) | [AGAATAATGGACGGTTCCTCTGG](https://www.maizegdb.org/data_center/primer?id=246332) |
| 121 | *umc1693* | 3.05 | (CCGG)4 | [CTTCGTCTTCCTCCACAAAACCTA](https://www.maizegdb.org/data_center/primer?id=292426) | [TGGTGATGCAGTAGTCGAGGC](https://www.maizegdb.org/data_center/primer?id=292427) |
| 122 | *phi073* | 3.05 | AGC | [GTGCGAGAGGCTTGACCAA](https://www.maizegdb.org/data_center/primer?id=111703) | [AAGGGTTGAGGGCGAGGAA](https://www.maizegdb.org/data_center/primer?id=111704) |
| 123 | *umc1102* | 3.05 | GGAT | [AAAATTAAAGTCAAGAGCGGGGAG](https://www.maizegdb.org/data_center/primer?id=235031) | [TCTAGCCGTAGCTTTAGCTGCATT](https://www.maizegdb.org/data_center/primer?id=235032) |
| 124 | *mmc0022* | 3.05 | (CA)6(CT)2(CA)6 | [AGGTGTTGTTTTTGTTCGCT](https://www.maizegdb.org/data_center/primer?id=167102) | [TGCTTGTTTAAGCTCATTATT](https://www.maizegdb.org/data_center/primer?id=167103) |
| 125 | *umc2020* | 3.05 | (GT)8 | [TTTGGAGTTATGGTTTGCGTTTTT](https://www.maizegdb.org/data_center/primer?id=309036) | [TGATAACGCTGAGAACGATGAAGA](https://www.maizegdb.org/data_center/primer?id=309037) |
| 126 | *umc1167* | 3.05 | (AG)12 | [CCTGCATGCATTAGGTATACGAAG](https://www.maizegdb.org/data_center/primer?id=235226) | [GTTTCTTCCAAGTTTTTGGCTTGA](https://www.maizegdb.org/data_center/primer?id=235227) |
| 127 | *umc2127* | 3.05 | (GGC)6 | [TATTACCTCTCTCCGCAGCTGTA](https://www.maizegdb.org/data_center/primer?id=484975) | [GATGCTTCTTCTCATCACCACTC](https://www.maizegdb.org/data_center/primer?id=484976) |
| 128 | *bnlg1035* | 3.05 | AG(13) | [TGCTTGCACTGTCAGGAATC](https://www.maizegdb.org/data_center/primer?id=171056) | [CAGCTCTGACACACCACACA](https://www.maizegdb.org/data_center/primer?id=171057) |
| 129 | *umc1644* | 3.06 | (TTG)8 | [CCATAAACTGTTCCTTTGGCACAC](https://www.maizegdb.org/data_center/primer?id=291265) | [CTTTCACGTGTTAAGGGAGACACC](https://www.maizegdb.org/data_center/primer?id=291266) |
| 130 | *umc2266* | 3.06 | (AAGGAG)4 | [GGACAGCTTGGCTTCGAGTG](https://www.maizegdb.org/data_center/primer?id=616206) | [ACGTTGGCCGTTAGTTCTTATCCT](https://www.maizegdb.org/data_center/primer?id=616207) |
| 131 | *umc1825* | 3.07 | (CT)7 | [ACTCAAGAGCAGACTGCAAAACCT](https://www.maizegdb.org/data_center/primer?id=301516) | [CGTGCATGTATTGTTTGTCCTAGC](https://www.maizegdb.org/data_center/primer?id=301517) |
| 132 | *umc1399* | 3.07 | (CTAG)5 | [GCTCTATGTTATTCTTCAATCGGGC](https://www.maizegdb.org/data_center/primer?id=256307) | [GGTCGGTCGGTACTCTGCTCTA](https://www.maizegdb.org/data_center/primer?id=256308) |
| 133 | *umc2174* | 3.08 | (CGA)4 | [GTACGTACGCAGCCACTTGTCAG](https://www.maizegdb.org/data_center/primer?id=485116) | [ACATAAATAAAACGTGTGCCGCAG](https://www.maizegdb.org/data_center/primer?id=485117) |
| 134 | *bnlg1108* | 3.08 | AG(21) | [GGATTCCTTTATGACGGGGT](https://www.maizegdb.org/data_center/primer?id=171108) | [AGTAACAACCAAGGCATCGG](https://www.maizegdb.org/data_center/primer?id=171109) |
| 135 | *umc2152* | 3.09 | (TG)8 | [TAGCTTCACCTGATGATCTTGCAC](https://www.maizegdb.org/data_center/primer?id=485050) | [CCTTTGTCTTCCGCTATCTTCCTT](https://www.maizegdb.org/data_center/primer?id=485051) |
| 136 | *umc1594* | 3.09 | (TA)10 | [GCCAGGGGAGAAATAAAATAAAGC](https://www.maizegdb.org/data_center/primer?id=273203) | [CACTGCAGGCCACACATACATA](https://www.maizegdb.org/data_center/primer?id=273204) |
| 137 | *umc1641* | 3.09 | (TCGCC)4 | [CTCCCTTCGTCTCCCGACTC](https://www.maizegdb.org/data_center/primer?id=291256) | [CAGATCGGCTCAGCCACAAC](https://www.maizegdb.org/data_center/primer?id=291257) |
| 138 | *umc2048* | 3.09 | (TC)6 | [GCTGAAGTCCCAACCACCAC](https://www.maizegdb.org/data_center/primer?id=309120) | [TTGACATGTTCTACCATCTCACCAA](https://www.maizegdb.org/data_center/primer?id=309121) |
| 139 | *bnlg1754* | 3.09 | AG(20) | [CCATCGCTGTACACATGAGG](https://www.maizegdb.org/data_center/primer?id=171460) | [TACCCGAAGGATCTGTTTGC](https://www.maizegdb.org/data_center/primer?id=171461) |
| 140 | *bnlg118* | 3.09 | AG(19) | [AGCCGAGTCAGTTCGAGGTA](https://www.maizegdb.org/data_center/primer?id=171168) | [CAGGGGCTTGAGGTGAGTTA](https://www.maizegdb.org/data_center/primer?id=171169) |
| 141 | *bnlg153* | 3.09 | AG(14) | [CAAAAAAAAAATATGTATACGGGG](https://www.maizegdb.org/data_center/primer?id=171342) | [ATGCACGAGCTTTTGGAGTT](https://www.maizegdb.org/data_center/primer?id=171343) |
| 142 | *bnlg1182* | 3.09 | AG(19) | [AGCCGAGTCAGTTCGAGGTA](https://www.maizegdb.org/data_center/primer?id=171168) | [CAGGGGCTTGAGGTGAGTTA](https://www.maizegdb.org/data_center/primer?id=171169) |
| 143 | *bnlg1496* | 3.09 | AG(18) | [CTGGGCAGACAGCAACAGTA](https://www.maizegdb.org/data_center/primer?id=171322) | [AGCCAAAGACATGATGGTCC](https://www.maizegdb.org/data_center/primer?id=171323) |
| 144 | *umc1232* | 4.00 | (ACAG)4 | [GGAATTACCACAACAAACTAAACTTGG](https://www.maizegdb.org/data_center/primer?id=242213) | [AGGCTCTAGCTACCTGGCTACGTT](https://www.maizegdb.org/data_center/primer?id=242214) |
| 145 | *umc2278* | 4.00 | (TCTC)4 | [CTGACCTCCGTCATCAGCATC](https://www.maizegdb.org/data_center/primer?id=616242) | [ATCACGGACAAAGAAAATTGAAGC](https://www.maizegdb.org/data_center/primer?id=616243) |
| 146 | *umc1008* | 4.00 | (GT)4(GA)6 | [TCTAGCTTGTGGTGGTGGTTGA](https://www.maizegdb.org/data_center/primer?id=167251) | [ACATGAGCACAAAGACTGACGC](https://www.maizegdb.org/data_center/primer?id=167252) |
| 147 | *umc1017* | 4.01 | (CT)9(CA)8 | [GAAGAGGTAAGGACGACGACGA](https://www.maizegdb.org/data_center/primer?id=167269) | [GCACCTGCAGTGAACGTCAGTA](https://www.maizegdb.org/data_center/primer?id=167270) |
| 148 | *umc1943* | 4.02 | NA | [GTGCTGCAGAATTCAACTCCTTC](https://www.maizegdb.org/data_center/primer?id=301870) | [ACCATTTCTGCGTTTCCACAGT](https://www.maizegdb.org/data_center/primer?id=301871) |
| 149 | *umc2281* | 4.03 | (GTCC)5 | [CAATGATTGGAGCCTAACCCCT](https://www.maizegdb.org/data_center/primer?id=616251) | [ATGATGATCTGCAGAGCCTAGTCC](https://www.maizegdb.org/data_center/primer?id=616252) |
| 150 | *nc004* | 4.03 | AG | [TGCGAAGAAGCAGTAGCAAA](https://www.maizegdb.org/data_center/primer?id=111617) | [TGGAGGTAGAAGACGCACG](https://www.maizegdb.org/data_center/primer?id=111618) |
| 151 | *umc2206* | 4.04 | (GTAC)4 | [CTCCTTCCCTTTCTCCTTCTGAAC](https://www.maizegdb.org/data_center/primer?id=616026) | [TTCACTTTTGCTTGTCGGCTG](https://www.maizegdb.org/data_center/primer?id=616027) |
| 152 | *bnlg490* | 4.04 | NA | [GCCCTAGCTTGCTAATTAACTAACA](https://www.maizegdb.org/data_center/primer?id=114522) | [ACTGTAAGGGCAGTGGACCTATA](https://www.maizegdb.org/data_center/primer?id=114523) |
| 153 | *phi026* | 4.05 | CT | [TAATTCCTCGCTCCCGGATTCAGC](https://www.maizegdb.org/data_center/primer?id=111657) | [GTGCATGAGGGAGCAGCAGGTAGTG](https://www.maizegdb.org/data_center/primer?id=111658) |
| 154 | *umc1511* | 4.05 | (CGA)4 | [CAGACAGATCCATCCAGCACATAC](https://www.maizegdb.org/data_center/primer?id=272954) | [GTTTGTAGGCTTCGTTTTCCTTCA](https://www.maizegdb.org/data_center/primer?id=272955) |
| 155 | *umc1662* | 4.05 | (AGCC)4 | [CCTTCTTCCTTCACGCCTCTTT](https://www.maizegdb.org/data_center/primer?id=291319) | [GACCACCTCATCTCTGACTCTGG](https://www.maizegdb.org/data_center/primer?id=291320) |
| 156 | *umc1088* | 4.05 | (CT)7 | [TCATCCTCCTAGCTCCTCTACTCG](https://www.maizegdb.org/data_center/primer?id=194033) | [AAAACAGTCAGCAGAACCCACTTT](https://www.maizegdb.org/data_center/primer?id=194034) |
| 157 | *bnlg1217* | 4.05 | AG(33) | [AGCTGATCTGCACGTTGTTG](https://www.maizegdb.org/data_center/primer?id=171190) | [GCAGATCCACGCCATTTAAA](https://www.maizegdb.org/data_center/primer?id=171191) |
| 158 | *bnlg1265* | 4.05 | AG(33) | [GGTTGTCCGTAAAGGCAAGA](https://www.maizegdb.org/data_center/primer?id=171212) | [TGTGAAGGCCAGACAGTCAG](https://www.maizegdb.org/data_center/primer?id=171213) |
| 159 | *umc1969* | 4.05 | NA | [CTCGAGCCCAGCAGAGAAAG](https://www.maizegdb.org/data_center/primer?id=301948) | [GGTGGAGCCCATGGCTATTACTAT](https://www.maizegdb.org/data_center/primer?id=301949) |
| 160 | *umc1390* | 4.05 | (CCT)4 | [CCTCGAAACAGATGCCTGAGTC](https://www.maizegdb.org/data_center/primer?id=256280) | [AAATGATCCCGAAGCCTGAGAC](https://www.maizegdb.org/data_center/primer?id=256281) |
| 161 | *umc2061* | 4.05 | (CTG)8 | [GTCTGGAGAACTCCCTACCCATTC](https://www.maizegdb.org/data_center/primer?id=309159) | [TAGCTTGAGAGACCGGAACAGC](https://www.maizegdb.org/data_center/primer?id=309160) |
| 162 | *nc005* | 4.05 | CT | [CCTCTACTCGCCAGTCGC](https://www.maizegdb.org/data_center/primer?id=111619) | [TTTGGTCAGATTTGAGCACG](https://www.maizegdb.org/data_center/primer?id=111620) |
| 163 | *phi079* | 4.05 | AGATG | [TGGTGCTCGTTGCCAAATCTACGA](https://www.maizegdb.org/data_center/primer?id=111715) | [GCAGTGGTGGTTTCGAACAGACAA](https://www.maizegdb.org/data_center/primer?id=111716) |
| 164 | *umc1303* | 4.05 | (CCG)4 | [CTTGGTAGCTTCGTATTCGACGAG](https://www.maizegdb.org/data_center/primer?id=246340) | [ATCCTAGGAAAGCAGGGAGGG](https://www.maizegdb.org/data_center/primer?id=246341) |
| 165 | *umc1964* | 4.05 | NA | [CTTCTCACTGTCGCAGAACAAGAG](https://www.maizegdb.org/data_center/primer?id=301933) | [CCGTATGTGTGTACTGTGGATTCAT](https://www.maizegdb.org/data_center/primer?id=301934) |
| 166 | *umc1451* | 4.05 | (GA)7 | [GGTAGATCGAGAAAGGAGTGGACA](https://www.maizegdb.org/data_center/primer?id=256430) | [TTGCAAGAGCACACGACTAAGAAG](https://www.maizegdb.org/data_center/primer?id=256431) |
| 167 | *umc1362* | 4.05 | (AGT)4 | [GATACCCACAGTGACCACGTTACA](https://www.maizegdb.org/data_center/primer?id=248685) | [GAAGGTGCAAAAGATGGACTCCTA](https://www.maizegdb.org/data_center/primer?id=248686) |
| 168 | *umc2054* | 4.05 | (GCA)5 | [CATTTCCTTCCCTGCTCTGATG](https://www.maizegdb.org/data_center/primer?id=309138) | [TACAGATACTGGAGCACTCTCGGC](https://www.maizegdb.org/data_center/primer?id=309139) |
| 169 | *umc1142* | 4.05 | (TGGA)5 | [CCGAAAACCCATTCTTCTAGCATC](https://www.maizegdb.org/data_center/primer?id=235151) | [GTGCGGTGTTCTCTCTTTCACTCT](https://www.maizegdb.org/data_center/primer?id=235152) |
| 170 | *umc1346* | 4.05 | (GCC)4 | [TCTGATCTCTTCGGTGCTAGAGAAA](https://www.maizegdb.org/data_center/primer?id=248637) | [AAGAGATCTCCCAACCCTAACTGC](https://www.maizegdb.org/data_center/primer?id=248638) |
| 171 | *umc1702* | 4.05 | (CAGCCT)4 | [ACGAGGCTCTTCCGAGTTCC](https://www.maizegdb.org/data_center/primer?id=292453) | [GTTTGAGGTGTTCACGGGTTCT](https://www.maizegdb.org/data_center/primer?id=292454) |
| 172 | *umc1869* | 4.06 | (GGT)6 | [CGAGCGCTCTAGACACGATTTT](https://www.maizegdb.org/data_center/primer?id=301648) | [GAACTGGAGGAGCGAGCATGTAT](https://www.maizegdb.org/data_center/primer?id=301649) |
| 173 | *bnlg1784* | 4.07 | AG(13) | [GCAACGATCTGTCAGACGAA](https://www.maizegdb.org/data_center/primer?id=171474) | [TTGGCATTGGTAATGGGTCT](https://www.maizegdb.org/data_center/primer?id=171475) |
| 174 | *dupssr34* | 4.07 | (TTG)14 | [TCAGTGCTTTCATTGTAACGA](https://www.maizegdb.org/data_center/primer?id=114212) | [ATAAACATCTTGCCAGCAAA](https://www.maizegdb.org/data_center/primer?id=114213) |
| 175 | *umc1620* | 4.07 | (TTC)4 | [CCACCGAGTGACTAGTTGTGAGAG](https://www.maizegdb.org/data_center/primer?id=273281) | [CCTTTCAATGTTCATGTTCTCTTCC](https://www.maizegdb.org/data_center/primer?id=273282) |
| 176 | *bnlg2162* | 4.08 | AG(27) | [GTCTGCTGCTAGTGGTGGTG](https://www.maizegdb.org/data_center/primer?id=171596) | [CACCGGCATTCGATATCTTT](https://www.maizegdb.org/data_center/primer?id=171597) |
| 177 | *umc1086* | 4.08 | (CT)12 | [CATGAAAGTTTTCCTGTGCAGATT](https://www.maizegdb.org/data_center/primer?id=194027) | GGGCAACTTTAGAGGTCGATTTATT |
| 178 | *umc1940* | 4.09 | NA | [AACAACAAATGGGATCTCCGTTAC](https://www.maizegdb.org/data_center/primer?id=301861) | [CCATCTGCTGAGGGCTTATCTG](https://www.maizegdb.org/data_center/primer?id=301862) |
| 179 | *umc1803* | 4.09 | (AG)32 | [TGACCTCTCCCTCTAGGCCTCTAC](https://www.maizegdb.org/data_center/primer?id=292756) | [CCCAATCTTCAGGTAATCCTCTCTC](https://www.maizegdb.org/data_center/primer?id=292757) |
| 180 | *umc1180* | 4.10 | (CATG)5 | [GAAGCCCCTTGAAATGAATGAAC](https://www.maizegdb.org/data_center/primer?id=235265) | [CGACGTACGTATAGACTCGCTCAG](https://www.maizegdb.org/data_center/primer?id=235266) |
| 181 | *umc1699* | 4.10 | (CA)8 | [CTTTTGCTCAAACACGGGAAATAC](https://www.maizegdb.org/data_center/primer?id=292444) | [AGGCATTGAGCGATATGTTTGTTT](https://www.maizegdb.org/data_center/primer?id=292445) |
| 182 | *phi076* | 4.11 | AGCGGG | [TTCTTCCGCGGCTTCAATTTGACC](https://www.maizegdb.org/data_center/primer?id=111709) | [GCATCAGGACCCGCAGAGTC](https://www.maizegdb.org/data_center/primer?id=111710) |
| 183 | *phi006* | 4.11 | CCT | [AGGCGGCGTGCTGAACACCT](https://www.maizegdb.org/data_center/primer?id=111633) | [CGCTTCATCTCCCGTGACAATG](https://www.maizegdb.org/data_center/primer?id=111634) |
| 184 | *umc1197* | 4.11 | AT | [GGTGTAATTTAGGGAGTGTTTGTTCG](https://www.maizegdb.org/data_center/primer?id=235316) | [CCGCATAGATGTGCTTTCTAGGAG](https://www.maizegdb.org/data_center/primer?id=235317) |
| 185 | *umc1050* | 4.11 | (AAT)6 | [CGATACACATCCATCTTCAGGTAGC](https://www.maizegdb.org/data_center/primer?id=193922) | [GCCTTTGTACCAATACAAGCCAAG](https://www.maizegdb.org/data_center/primer?id=193923) |
| 186 | *umc1649* | 4.11 | (CA)6 | [GTGAAGCTCGATTTCTCCTCACAT](https://www.maizegdb.org/data_center/primer?id=291280) | [CAGCGACACCATGATAAGTACACC](https://www.maizegdb.org/data_center/primer?id=291281) |
| 187 | *umc2290* | 4.11 | (CTT)4 | [GAATCACAATGGATCCTACCAAGG](https://www.maizegdb.org/data_center/primer?id=616278) | [AACTGCAAGCAAAATCAACACAAG](https://www.maizegdb.org/data_center/primer?id=616279) |
| 188 | *umc1253* | 5.00 | (TTC)4 | [GAGGTAGGCGTCGTATGCTCTAAA](https://www.maizegdb.org/data_center/primer?id=242276) | [AACGTGACTTACAAGGTTGCGTTC](https://www.maizegdb.org/data_center/primer?id=242277) |
| 189 | *umc1097* | 5.00 | (CA)8 | [CTCGTCAACGTCAACCCAAGTAAG](https://www.maizegdb.org/data_center/primer?id=194060) | [CTGTTAGATGTGCGACAACAGAGC](https://www.maizegdb.org/data_center/primer?id=194061) |
| 190 | *umc1491* | 5.00 | (AGA)5 | [TAATAATCCCAAACCACCAAAAGG](https://www.maizegdb.org/data_center/primer?id=272894) | [GATTTGAGGCCATAGTGCTCCTTA](https://www.maizegdb.org/data_center/primer?id=272895) |
| 191 | *umc1308* | 5.00 | (TG)10 | [GCAGATGGACACAAACAAATGAAG](https://www.maizegdb.org/data_center/primer?id=246355) | [GCTACTGATGCTGGCAATCTTACA](https://www.maizegdb.org/data_center/primer?id=246356) |
| 192 | *umc1365* | 5.01 | (TAC)5 | [GCATACATGATACATGCCGTGACT](https://www.maizegdb.org/data_center/primer?id=248694) | [TGCACACAGTAGGAAGACAGGAAG](https://www.maizegdb.org/data_center/primer?id=248695) |
| 193 | *bnlg1836* | 5.01 | AG(12) | [GGGTTGATGCAAGATGGAAC](https://www.maizegdb.org/data_center/primer?id=171506) | [AGACGAAACATACGAACGGG](https://www.maizegdb.org/data_center/primer?id=171507) |
| 194 | *umc1935* | 5.03 | (CA)8 | [TGTGCTGTGTGTAATATGGTCGTG](https://www.maizegdb.org/data_center/primer?id=301846) | [TAATCAGGAGGAGAATTCGGTCAA](https://www.maizegdb.org/data_center/primer?id=301847) |
| 195 | *umc1705* | 5.03 | (AG)28 | [ATCTCACGTACGGTAATGCAGACA](https://www.maizegdb.org/data_center/primer?id=292462) | [CATGACCTGATAAACCCTCCTCTC](https://www.maizegdb.org/data_center/primer?id=292463) |
| 196 | *bnlg1046* | 5.03 | AG(39) | [TGAGCCGAAGCTAACCTCTC](https://www.maizegdb.org/data_center/primer?id=171070) | [GATGCAAAGGAGGTTCAGGA](https://www.maizegdb.org/data_center/primer?id=171071) |
| 197 | *bnlg2323* | 5.04 | AG(25) | [ACCGTCTCAGCAAAATGGTC](https://www.maizegdb.org/data_center/primer?id=171644) | [CCGCCTTCACTATGGTCAAT](https://www.maizegdb.org/data_center/primer?id=171645) |
| 198 | *phi333597* | 5.05 | AAG | [AGCTCGAGTACCTGCCGAG](https://www.maizegdb.org/data_center/primer?id=256163) | [TGCATCTCTGAGACCATCACC](https://www.maizegdb.org/data_center/primer?id=256164) |
| 199 | *phi085* | 5.06 | AACGC | [AGCAGAACGGCAAGGGCTACT](https://www.maizegdb.org/data_center/primer?id=111727) | [TTTGGCACACCACGACGA](https://www.maizegdb.org/data_center/primer?id=111728) |
| 200 | *bnlg1118* | 5.07 | AG(15) | [CAGAGTTGATGAACTGAAAAAGG](https://www.maizegdb.org/data_center/primer?id=171116) | [CTCTTGCTTCCCCCCTAATC](https://www.maizegdb.org/data_center/primer?id=171117) |
| 201 | *bnlg1346* | 5.07 | AG(24) | [CATCATGAAGCAATGAAGCC](https://www.maizegdb.org/data_center/primer?id=171256) | [CCGCGCCATTATCTAGTTGT](https://www.maizegdb.org/data_center/primer?id=171257) |
| 202 | *bnlg1885* | 5.07 | AG(23) | [GACAGACGCAACTACCGAAA](https://www.maizegdb.org/data_center/primer?id=171522) | [TGTTCAATTTGATGTTCATTGC](https://www.maizegdb.org/data_center/primer?id=171523) |
| 203 | *umc2143* | 5.08 | (TTC)4 | [ACACACAACAGAGCCTTTTGTTCA](https://www.maizegdb.org/data_center/primer?id=485023) | [AAGAAAAGGACACCAAACCAAACA](https://www.maizegdb.org/data_center/primer?id=485024) |
| 204 | *umc2136* | 5.08 | (CCT)8 | [CCAGATGCGGAAGTAGACGG](https://www.maizegdb.org/data_center/primer?id=485002) | [GATTCGGAGGTGATCTGACCTGT](https://www.maizegdb.org/data_center/primer?id=485003) |
| 205 | *umc1829* | 5.09 | (AG)10 | [GTTGATTGGTTGATGTGGAAACAA](https://www.maizegdb.org/data_center/primer?id=301528) | [CAGTTTGATGTTCATGGCTCTCTC](https://www.maizegdb.org/data_center/primer?id=301529) |
| 206 | *umc2307* | 5.09 | (CAG)4 | [GTCGACATCGTCTTCCCCAAG](https://www.maizegdb.org/data_center/primer?id=616329) | [GTAGGAAGCCACGTACGGCTC](https://www.maizegdb.org/data_center/primer?id=616330) |
| 207 | *bnlg1902* | 5.30 | AG(12) | [AACTACCGTCGAAGTGGTGG](https://www.maizegdb.org/data_center/primer?id=171532) | [CGCCTCTCTCTGACTTGTTG](https://www.maizegdb.org/data_center/primer?id=171533) |
| 208 | *umc1143* | 6.00 | AAAAT | [GACACTAGCAATGTTCAAAACCCC](https://www.maizegdb.org/data_center/primer?id=235154) | [CGTGGTGGGATGCTATCCTTT](https://www.maizegdb.org/data_center/primer?id=235155) |
| 209 | *umc1753* | 6.00 | (CT)7 | [AAGATCTTGCTCCGTTTCCTCTCT](https://www.maizegdb.org/data_center/primer?id=292606) | [TTCAGATGCAAATCTCTTTTCGCT](https://www.maizegdb.org/data_center/primer?id=292607) |
| 210 | *bnlg1867* | 6.01 | AG(17) | [CCACCACCATCGTAGGAGTT](https://www.maizegdb.org/data_center/primer?id=171516) | [CAGTACACAGCAGGCAGCTC](https://www.maizegdb.org/data_center/primer?id=171517) |
| 211 | *bnlg1422* | 6.01 | AG(22) | [GACGATTAACAGGTGGGGAC](https://www.maizegdb.org/data_center/primer?id=171288) | [ATGATGCAAATGAGGCACAA](https://www.maizegdb.org/data_center/primer?id=171289) |
| 212 | *umc1376* | 6.01 | (AGC)6 | [GAGGACGAGGAGGAAGACGAGAT](https://www.maizegdb.org/data_center/primer?id=256238) | [CATGGGAACGTGCTCCACAC](https://www.maizegdb.org/data_center/primer?id=256239) |
| 213 | *umc1014* | 6.04 | (AG)12 | [GAAAGTCGATCGAGAGACCCTG](https://www.maizegdb.org/data_center/primer?id=167263) | [CCCTCTCTTCACCCCTTCCTT](https://www.maizegdb.org/data_center/primer?id=167264) |
| 214 | *umc2006* | 6.04 | NA | [AGTCCATCACCATCCCTGGC](https://www.maizegdb.org/data_center/primer?id=302059) | [GCAGAACTATTGTCAGTTAACCTTGCAT](https://www.maizegdb.org/data_center/primer?id=302060) |
| 215 | *umc1979* | 6.04 | NA | [AATTTCGGGAAACAGGCCAT](https://www.maizegdb.org/data_center/primer?id=301978) | [GAGTCCCCGAAACTGAACACC](https://www.maizegdb.org/data_center/primer?id=301979) |
| 216 | *umc1918* | 6.04 | (AT)8 | [CACAAGAACATTATGACGACCGAG](https://www.maizegdb.org/data_center/primer?id=301795) | [AAGCAGGAGACATCGTTTAAGTCG](https://www.maizegdb.org/data_center/primer?id=301796) |
| 217 | *umc1105* | 6.04 | (GCC)4 | [ATTCCTGCATCATCATCCACTACA](https://www.maizegdb.org/data_center/primer?id=235040) | [GCCAACTGATCTGCTCTAGCTTC](https://www.maizegdb.org/data_center/primer?id=235041) |
| 218 | *umc2320* | 6.05 | (GGT)4 | [TAACCTCTAGCAGCACATGCACAC](https://www.maizegdb.org/data_center/primer?id=616368) | [GAGAGTTTTATCAGCAGCAAAGCC](https://www.maizegdb.org/data_center/primer?id=616369) |
| 219 | *umc1826* | 6.05 | (ATC)6 | [CTGGCTTAGGAGAACTTCCTCATT](https://www.maizegdb.org/data_center/primer?id=301519) | [TGATAATCTTGATGATCTCGGTGG](https://www.maizegdb.org/data_center/primer?id=301520) |
| 220 | *bnlg2249* | 6.05 | AG(20) | [AGGATCCCCTAGCAAAAGGA](https://www.maizegdb.org/data_center/primer?id=171628) | [CCCCCTAGTTCGTTGCATAA](https://www.maizegdb.org/data_center/primer?id=171629) |
| 221 | *umc1114* | 6.05 | (AGAA)6 | [CAATGTGTTATTGATTGTACACCGC](https://www.maizegdb.org/data_center/primer?id=235067) | [ACAGCAGGAGGCAGAGACTGAC](https://www.maizegdb.org/data_center/primer?id=235068) |
| 222 | *umc2141* | 6.05 | (CT)8 | [ATTAGCACCACCGTGTAGCAAGTT](https://www.maizegdb.org/data_center/primer?id=485017) | [GGCAGTGTGAGTGGTTGTGTG](https://www.maizegdb.org/data_center/primer?id=485018) |
| 223 | *umc2322* | 6.06 | (GCG)6 | [AACAAGTGGCGACAGACAAAGATT](https://www.maizegdb.org/data_center/primer?id=616374) | [CTGCAGGTCCCTGAGCCTCT](https://www.maizegdb.org/data_center/primer?id=616375) |
| 224 | *umc1859* | 6.06 | (TC)8 | [ATATACATGTGAGCTGGTTGCCCT](https://www.maizegdb.org/data_center/primer?id=301618) | [GCATGCTATTACCAATCTCCAGGT](https://www.maizegdb.org/data_center/primer?id=301619) |
| 225 | *umc1762* | 6.06 | (TC)7 | [CTTACTCCAGGCACTCCATACCAT](https://www.maizegdb.org/data_center/primer?id=292633) | [ATCCAGGTGAATGGTGTTTACGAT](https://www.maizegdb.org/data_center/primer?id=292634) |
| 226 | *umc1520* | 6.06 | (GA)8 | [AGCAAATATATGAGCAATTAAGAACAGG](https://www.maizegdb.org/data_center/primer?id=272981) | [GTGTCGCCACCTATAATTTGATGA](https://www.maizegdb.org/data_center/primer?id=272982) |
| 227 | *umc2375* | 6.06 | (GCG)4 | [GCCGTACTGATGTGATGGTCC](https://www.maizegdb.org/data_center/primer?id=616533) | [TCTGACATTGTCCTCTTGACCAAA](https://www.maizegdb.org/data_center/primer?id=616534) |
| 228 | *umc1248* | 6.07 | (TC)12 | [CTTTGTCCATCGGCTTTATTCTTT](https://www.maizegdb.org/data_center/primer?id=242261) | [CACATTAAGTTACAAATACAAATCACCG](https://www.maizegdb.org/data_center/primer?id=242262) |
| 229 | *umc1490* | 6.07 | (AC)6 | [AAGAAAGGGTTTCGGGGATAAAC](https://www.maizegdb.org/data_center/primer?id=272891) | [ATTCGGCTGTATATGATCCGATGT](https://www.maizegdb.org/data_center/primer?id=272892) |
| 230 | *umc1779* | 6.07 | (TCG)4 | [AGGAGTACGGTCGGCACGTT](https://www.maizegdb.org/data_center/primer?id=292684) | [GCTGCTGCTGGTTGTGATAGC](https://www.maizegdb.org/data_center/primer?id=292685) |
| 231 | *bnlg1521* | 6.07 | AG(27) | [GTTGCATACACACCACAGACA](https://www.maizegdb.org/data_center/primer?id=171334) | [GATACCTTCCCTGCCTCACA](https://www.maizegdb.org/data_center/primer?id=171335) |
| 232 | *hir3* | 6.08 | NA | GATCCTCTGTCGCCAAACACTAAG | AGATGGTGACGATGAGTGATGAAC |
| 233 | *umc1642* | 7.00 | (GCTA)6 | [CACTACAGCGCCTGTAACTGCC](https://www.maizegdb.org/data_center/primer?id=291259) | [CATGAGCTAAGCAAGAGGGGTATG](https://www.maizegdb.org/data_center/primer?id=291260) |
| 234 | *umc1788* | 7.00 | (AAAAT)4 | [CACTAATTGCTACCAGGAGATTCAAA](https://www.maizegdb.org/data_center/primer?id=292711) | [AAGTCAGTGAAGCGCTGAGTTTTT](https://www.maizegdb.org/data_center/primer?id=292712) |
| 235 | *umc1426* | 7.00 | (AGAGG)4 | [TAGGGTCGATTCTGGATTGTCTG](https://www.maizegdb.org/data_center/primer?id=256385) | [TGTAAAACAGAAAGCATGCGAGTC](https://www.maizegdb.org/data_center/primer?id=256386) |
| 236 | *umc1480* | 7.00 | (GAA)4 | [AATGAAGGTGGATGTGCTGCTACT](https://www.maizegdb.org/data_center/primer?id=256517) | [CTTCCCCATCTCCTCTTGAAGATT](https://www.maizegdb.org/data_center/primer?id=256518) |
| 237 | *umc2177* | 7.00 | (GTC)4 | [ACCATGCATGTCTCACGTCACT](https://www.maizegdb.org/data_center/primer?id=485125) | [GGGTACGTGCTGTGGAGGAC](https://www.maizegdb.org/data_center/primer?id=485126) |
| 238 | *bnlg1367* | 7.00 | AG(42) | [CGACGGCGTACAGAGAGAG](https://www.maizegdb.org/data_center/primer?id=171266) | [GGTCGCCACCCCACCT](https://www.maizegdb.org/data_center/primer?id=171267) |
| 239 | *umc1545* | 7.00 | (AAGA)4 | [GAAAACTGCATCAACAACAAGCTG](https://www.maizegdb.org/data_center/primer?id=273056) | [ATTGGTTGGTTCTTGCTTCCATTA](https://www.maizegdb.org/data_center/primer?id=273057) |
| 240 | *umc1583* | 7.00 | (GAA)4 | [AAAGGGCGACTTGTTTTTGTTTTT](https://www.maizegdb.org/data_center/primer?id=273170) | [GCCTGCTTTTGTGTATCTTAGGCA](https://www.maizegdb.org/data_center/primer?id=273171) |
| 241 | *umc2160* | 7.01 | (AG)10 | [TAAAACCTTTACCCCATCCAGCAT](https://www.maizegdb.org/data_center/primer?id=485074) | [TGTGCTCGTGCTTCTCTCTGAGTA](https://www.maizegdb.org/data_center/primer?id=485075) |
| 242 | *umc1428* | 7.01 | (CCG)5 | [CTATCTCGGTAACTCCCACCAAAG](https://www.maizegdb.org/data_center/primer?id=256391) | [GTTACTACCGTGATGAAACGAGGG](https://www.maizegdb.org/data_center/primer?id=256392) |
| 243 | *bnlg1022* | 7.02 | AG(12) | [GTGTTGTCGATCCACTCCCT](https://www.maizegdb.org/data_center/primer?id=171046) | [GCAAAGATCTGTGAGGGGAC](https://www.maizegdb.org/data_center/primer?id=171047) |
| 244 | *umc1036* | 7.02 | GA COMPLEX | [CTGCTGCTCAAGGAGATGGAGA](https://www.maizegdb.org/data_center/primer?id=174773) | [GACACACATGCACGAGCAGACT](https://www.maizegdb.org/data_center/primer?id=174774) |
| 245 | *phi034* | 7.02 | CCT | [TAGCGACAGGATGGCCTCTTCT](https://www.maizegdb.org/data_center/primer?id=111671) | [GGGGAGCACGCCTTCGTTCT](https://www.maizegdb.org/data_center/primer?id=111672) |
| 246 | *umc1879* | 7.02 | (GC)6 | [TTTCTGATTAGCTAGAAGCGCGAG](https://www.maizegdb.org/data_center/primer?id=301678) | [GATAAAACAGTACTGGGCCGATTG](https://www.maizegdb.org/data_center/primer?id=301679) |
| 247 | *umc1068* | 7.02 | (GAAAA)6(GAA)2 | [AGTCGTTTTCAAAGGCTGCTGATA](https://www.maizegdb.org/data_center/primer?id=193974) | [TGAGTCACCTCATTTCTTCTGGTTC](https://www.maizegdb.org/data_center/primer?id=193975) |
| 248 | *bnlg1759* | 7.02 | AG(23) | [AGACGGAGTCCTCGTTTGC](https://www.maizegdb.org/data_center/primer?id=171464) | [ACCGGTTCGTACCACTCACT](https://www.maizegdb.org/data_center/primer?id=171465) |
| 249 | *bnlg2203* | 7.02 | AG(22) | [CTCCGGCGAGCCCAGAC](https://www.maizegdb.org/data_center/primer?id=171608) | [CTCGACCCATGCTCTCCTCT](https://www.maizegdb.org/data_center/primer?id=171609) |
| 250 | *bnlg1003* | 7.02 | AG(12) | [GACCCTCAACCGACCAGTAA](https://www.maizegdb.org/data_center/primer?id=171028) | [ATGGCCTTTTTAAAGGAGGG](https://www.maizegdb.org/data_center/primer?id=171029) |
| 251 | *bnlg1094* | 7.02 | AG(21) | [GTGAAGAACGATGACGCAGA](https://www.maizegdb.org/data_center/primer?id=171104) | [CAGCAACGCTCTCACATTGT](https://www.maizegdb.org/data_center/primer?id=171105) |
| 252 | *bnlg1380* | 7.02 | AG(15) | [ACAATTCGATCGAGAGCGAG](https://www.maizegdb.org/data_center/primer?id=171276) | [CCTTTCTTGCTGGTTCTTGC](https://www.maizegdb.org/data_center/primer?id=171277) |
| 253 | *umc1837* | 7.03 | (TA)8 | [AAGTGTTGGGAAAGAAGGGGACT](https://www.maizegdb.org/data_center/primer?id=301552) | [TGGTCGGGGCTTCCTATAGTTAAA](https://www.maizegdb.org/data_center/primer?id=301553) |
| 254 | *umc1567* | 7.03 | (AGA)4 | [GCGGCAGGAGTACTCACTATATGC](https://www.maizegdb.org/data_center/primer?id=273122) | [GTCCGAGAATAAGATCGTTGATGG](https://www.maizegdb.org/data_center/primer?id=273123) |
| 255 | *umc1987* | 7.03 | NA | [ACCCTCCGAAAAGCAAGCTC](https://www.maizegdb.org/data_center/primer?id=302002) | [CGTGGGCTCCTCCTTCTTGT](https://www.maizegdb.org/data_center/primer?id=302003) |
| 256 | *bnlg1070* | 7.03 | AG(15) | [TTCCAGTAAGGGAGGTGCTG](https://www.maizegdb.org/data_center/primer?id=171088) | [TAAGCAACATATAGCCGGGC](https://www.maizegdb.org/data_center/primer?id=171089) |
| 257 | *umc1275* | 7.03 | (AG)6 | [GTTCGCAGGCCTAGACTCTAATGA](https://www.maizegdb.org/data_center/primer?id=246256) | [AGTTCTAGTCCCTGGTGATCCTCC](https://www.maizegdb.org/data_center/primer?id=246257) |
| 258 | *phi328175* | 7.04 | AGG | [GGGAAGTGCTCCTTGCAG](https://www.maizegdb.org/data_center/primer?id=256151) | [CGGTAGGTGAACGCGGTA](https://www.maizegdb.org/data_center/primer?id=256152) |
| 259 | *umc1251* | 7.04 | (ATC)4 | [GGTAGTGCTTGGTATTGACATCAGA](https://www.maizegdb.org/data_center/primer?id=242270) | [CTCTTGAAGATGGTCCTAGCATTG](https://www.maizegdb.org/data_center/primer?id=242271) |
| 260 | *umc1342* | 7.04 | (AG)8 | [TCTAATCCAATCGACATCGACAGA](https://www.maizegdb.org/data_center/primer?id=248625) | [TCGCCCTCTTTTCTTTTCTTTTCT](https://www.maizegdb.org/data_center/primer?id=248626) |
| 261 | *dupssr13* | 7.04 | (CA)12 | [TCGTTCGGTCCATGAAAT](https://www.maizegdb.org/data_center/primer?id=114166) | [CAAATATCTCTCATCTTTGCTGAC](https://www.maizegdb.org/data_center/primer?id=114167) |
| 262 | *umc2368* | 7.05 | (GCT)4 | [CGCCCCTAAGCTTTTATTTCTGTT](https://www.maizegdb.org/data_center/primer?id=616512) | [GCAATTGCTCCTTTTCCTTTTCTT](https://www.maizegdb.org/data_center/primer?id=616513) |
| 263 | *phi069* | 7.05 | GAC | [AGACACCGCCGTGGTCGTC](https://www.maizegdb.org/data_center/primer?id=130855) | [AGTCCGGCTCCACCTCCTTC](https://www.maizegdb.org/data_center/primer?id=130856) |
| 264 | *bnlg1073* | 8.01 | AG(19) | [TCGATCTAAGTATTGTAAACGTACG](https://www.maizegdb.org/data_center/primer?id=171090) | [GTATTTGGAGGCGCCATAGA](https://www.maizegdb.org/data_center/primer?id=171091) |
| 265 | *umc1592* | 8.01 | CA | [GACCATATGTGCTCCAAAACCTTC](https://www.maizegdb.org/data_center/primer?id=273197) | [AAGCTTCTTCGGTCTTTGTAGGGT](https://www.maizegdb.org/data_center/primer?id=273198) |
| 266 | *umc1790* | 8.02 | (AG)7 | [GAGAGGGAGGAGCAAGGAAATAGA](https://www.maizegdb.org/data_center/primer?id=292717) | GAGAAGAGTTCTCCATGGAACGAG |
| 267 | *umc2352* | 8.02 | (AAAG)6 | [ACAGACCTATGTGGCTATGCCTTC](https://www.maizegdb.org/data_center/primer?id=616464) | [ACGAAGCTAGGAAAGAGGCATTCT](https://www.maizegdb.org/data_center/primer?id=616465) |
| 268 | *bnlg1352* | 8.02 | AG(16) | [AAACAAAGCAGAGAGCGGAA](https://www.maizegdb.org/data_center/primer?id=171262) | [CCGTCCGTCTGCTGTAAATT](https://www.maizegdb.org/data_center/primer?id=171263) |
| 269 | *umc1778* | 8.03 | (GTC)6 | [GTGAACCATTGTAGCTGTCCCTG](https://www.maizegdb.org/data_center/primer?id=292681) | [GAGCTCGTACCTGTTCATGAGGAT](https://www.maizegdb.org/data_center/primer?id=292682) |
| 270 | *phi014* | 8.04 | GGC | [AGATGACCAGGGCCGTCAACGAC](https://www.maizegdb.org/data_center/primer?id=111639) | [CCAGCTTCACCAGCTTGCTCTTCGTG](https://www.maizegdb.org/data_center/primer?id=111640) |
| 271 | *umc1858* | 8.04 | (TA)8 | [GTTGTTCTCCTTGCTGACCAGTTT](https://www.maizegdb.org/data_center/primer?id=301615) | [ATCAGCAAATTAAAGCAAAGGCAG](https://www.maizegdb.org/data_center/primer?id=301616) |
| 272 | *umc2367* | 8.05 | (CCG)6 | [AACCGTTGTCGTTGTCTCTGTTC](https://www.maizegdb.org/data_center/primer?id=616509) | [ATGAACTTGGTCACGCTCATCTC](https://www.maizegdb.org/data_center/primer?id=616510) |
| 273 | *umc1828* | 8.06 | (GA)8 | [ACATGACCAAGCCATCATTCTACA](https://www.maizegdb.org/data_center/primer?id=301525) | [CCCTTTCCATCTTTATCCCTCATC](https://www.maizegdb.org/data_center/primer?id=301526) |
| 274 | *umc1724* | 8.06 | (CGA)4 | [GTCTCAAGTGAAACAACCACGCTT](https://www.maizegdb.org/data_center/primer?id=292519) | [CCACATGAGATGAGATTGCCATT](https://www.maizegdb.org/data_center/primer?id=292520) |
| 275 | *phi015* | 8.08 | AAAC | [GCAACGTACCGTACCTTTCCGA](https://www.maizegdb.org/data_center/primer?id=111641) | [ACGCTGCATTCAATTACCGGGAAG](https://www.maizegdb.org/data_center/primer?id=111642) |
| 276 | *umc1933* | 8.08 | (CCA)6 | [CGGAAAGACATGAAGCACACTATG](https://www.maizegdb.org/data_center/primer?id=301840) | [CACGGAGAAAGTGGGTCTATATGC](https://www.maizegdb.org/data_center/primer?id=301841) |
| 277 | *umc1673* | 8.08 | (TCC)5 | [AAGCTCAAGCTCCTAGCTCTTCCT](https://www.maizegdb.org/data_center/primer?id=291352) | [GAGGAGCGTCTCCAGAAGGAC](https://www.maizegdb.org/data_center/primer?id=291353) |
| 278 | *umc1279* | 9.00 | (CCT)6 | [GATGAGCTTGACGACGCCTG](https://www.maizegdb.org/data_center/primer?id=246268) | [CAATCCAATCCGTTGCAGGTC](https://www.maizegdb.org/data_center/primer?id=246269) |
| 279 | *bnlg2122* | 9.01 | AG(17) | [TCATCTGGCAAAACCTAGCC](https://www.maizegdb.org/data_center/primer?id=171582) | [CTTGCCAACTTGAGGACATG](https://www.maizegdb.org/data_center/primer?id=171583) |
| 280 | *bnlg1724* | 9.01 | AG(31) | [CTGACCCAGAGCATTGTGAA](https://www.maizegdb.org/data_center/primer?id=171444) | [GATGAAGAGCTTGCAGTCCC](https://www.maizegdb.org/data_center/primer?id=171445) |
| 281 | *bnlg1372* | 9.02 | AG(14) | [AGCGGTGCTCAAATAGGAG](https://www.maizegdb.org/data_center/primer?id=171272) | [CGCCGGCTTCCCTCAC](https://www.maizegdb.org/data_center/primer?id=171273) |
| 282 | *phi017* | 9.02 | TAC | [CGTTGGCGACCAGGGTGCGTTGGAT](https://www.maizegdb.org/data_center/primer?id=111645) | [TGCAACAGCCATTCGATCATCAAAC](https://www.maizegdb.org/data_center/primer?id=111646) |
| 283 | *bnlg1401* | 9.02 | AG(22) | [CACTCGGTTTTTGCTTAGCC](https://www.maizegdb.org/data_center/primer?id=171284) | [GTGTCGTCGAGTGCATGC](https://www.maizegdb.org/data_center/primer?id=171285) |
| 284 | *umc1170* | 9.02 | (TC)12 | [TGGGTGCTAAAACGTAACAACAAA](https://www.maizegdb.org/data_center/primer?id=235235) | [GAGGACGAAGCAGAAATCCTACC](https://www.maizegdb.org/data_center/primer?id=235236) |
| 285 | *umc1764* | 9.02 | (GGC)4 | [AATAAGCATGCTGCTACTGTCGTG](https://www.maizegdb.org/data_center/primer?id=292639) | [ACGCATACACTTCCTTATTGGCAT](https://www.maizegdb.org/data_center/primer?id=292640) |
| 286 | *umc1634* | 9.03 | (AG)7 | [TCCGTTGAGGACACTCGAATTTAT](https://www.maizegdb.org/data_center/primer?id=291235) | [GTAGCCTGCAAAACATCCAAGAAC](https://www.maizegdb.org/data_center/primer?id=291236) |
| 287 | *bnlg1159* | 9.04 | AG(15) | [GTGTGCCTATCCTTCCGAGA](https://www.maizegdb.org/data_center/primer?id=171144) | [AAGGACGTCAACAACGAACC](https://www.maizegdb.org/data_center/primer?id=171145) |
| 288 | *bnlg1209* | 9.04 | AG(12) | [GTCCCGGGCAGAATAATACC](https://www.maizegdb.org/data_center/primer?id=171188) | [TTCCTCCTTGAAGTGCTCGT](https://www.maizegdb.org/data_center/primer?id=171189) |
| 289 | *bnlg1012* | 9.04 | AG(16) | [GAGTGAGCGTGCGGAGTC](https://www.maizegdb.org/data_center/primer?id=171034) | [AACAGGCCAAACTCCTCCTC](https://www.maizegdb.org/data_center/primer?id=171035) |
| 290 | *umc1878* | 9.04 | (AT)6 | [GACCTTAAGTGCCAAACGTCACAT](https://www.maizegdb.org/data_center/primer?id=301675) | [GCTAATGATCGGGTGGAAAGTATG](https://www.maizegdb.org/data_center/primer?id=301676) |
| 291 | *phi016* | 9.04 | GGT | [TTCCATCATTGATCCGGGTGTCG](https://www.maizegdb.org/data_center/primer?id=111643) | [AAGGAGCAACATCCCATCCAGGAA](https://www.maizegdb.org/data_center/primer?id=111644) |
| 292 | *umc2121* | 9.04 | (AGCG)4 | [AAAAGTGGGCGCTAGTACAAGATG](https://www.maizegdb.org/data_center/primer?id=484957) | [GTGGTGTTCTCCTTTTGATACGCT](https://www.maizegdb.org/data_center/primer?id=484958) |
| 293 | *umc1357* | 9.05 | (CTG)8 | [TAGACATGTTGAAACCAGGACCG](https://www.maizegdb.org/data_center/primer?id=248670) | [ACGACGTCAACAACAGCATGA](https://www.maizegdb.org/data_center/primer?id=248671) |
| 294 | *umc1078* | 9.05 | (GT)13 | [AGGCACTAGCAGGCGAGAGG](https://www.maizegdb.org/data_center/primer?id=194003) | [GCGTAGTAACATCCATCCAACCAA](https://www.maizegdb.org/data_center/primer?id=194004) |
| 295 | *umc2341* | 9.05 | (TTC)6 | [CTGAGCTCCTGATTTCTTGCTCTC](https://www.maizegdb.org/data_center/primer?id=616431) | [AAACATTTAATCCAACAGCCCAGA](https://www.maizegdb.org/data_center/primer?id=616432) |
| 296 | *umc2338* | 9.05 | (GCC)4 | [ATCTCACACTCACGACCTGTTGTC](https://www.maizegdb.org/data_center/primer?id=616422) | [AGCATTGGGACTGTTCAAGAGAAG](https://www.maizegdb.org/data_center/primer?id=616423) |
| 297 | *umc1231* | 9.05 | (GA)10 | [CTGTAGGGCTGAGAAAAGAGAGGG](https://www.maizegdb.org/data_center/primer?id=248529) | [CGACAACTTAGGAGAACCATGGAG](https://www.maizegdb.org/data_center/primer?id=248530) |
| 298 | *umc2344* | 9.05 | (GGCGGT)4 | [CCGTTGTAGGGGGCGTAGTC](https://www.maizegdb.org/data_center/primer?id=616440) | [ATCCACCACCTCGACGCTTT](https://www.maizegdb.org/data_center/primer?id=616441) |
| 299 | *bnlg292* | 9.06 | NA | [TGGTAGGACCTTACAATGGGA](https://www.maizegdb.org/data_center/primer?id=114459) | [CGGGAGTACTGCTACACACGA](https://www.maizegdb.org/data_center/primer?id=114460) |
| 300 | *umc1366* | 9.06 | (TCC)4 | [GTCACTCGTCCGCATCGTCT](https://www.maizegdb.org/data_center/primer?id=248697) | [CCTAACTCTGCAAAGACTGCATGA](https://www.maizegdb.org/data_center/primer?id=248698) |
| 301 | *phi448880* | 9.06 | AAG | [CGATCCGGAGGAGTTCCTTA](https://www.maizegdb.org/data_center/primer?id=256211) | [CCATGAACATGCCAATGC](https://www.maizegdb.org/data_center/primer?id=256212) |
| 302 | *bnlg128* | 9.07 | NA | [CACCTGGAGGGACCCATTCC](https://www.maizegdb.org/data_center/primer?id=114546) | [AGGACCACAGGATCCATCATCCT](https://www.maizegdb.org/data_center/primer?id=114547) |
| 303 | *umc1137* | 9.08 | (CT)15 | [ATCAGTCACTCTTCTGCCTCCACT](https://www.maizegdb.org/data_center/primer?id=235136) | [GGCTGGATAATGTTGTAGCTGGTC](https://www.maizegdb.org/data_center/primer?id=235137) |
| 304 | *umc1505* | 9.08 | (AAAAC)4 | [TTACACAGAAGCCCATTTGAAGGT](https://www.maizegdb.org/data_center/primer?id=272936) | [GGATGGTTGTTGGTGGTGTAGAAT](https://www.maizegdb.org/data_center/primer?id=272937) |
| 305 | *umc2528* | 10.00 | AT | CTCATCAACATGCAAAGGACGTAG | ATTCAAATGCCTCTAAGCTAGCCG |
| 306 | *phi041* | 10.00 | AGCC | [TTGGCTCCCAGCGCCGCAAA](https://www.maizegdb.org/data_center/primer?id=130825) | [GATCCAGAGCGATTTGACGGCA](https://www.maizegdb.org/data_center/primer?id=130826) |
| 307 | *umc1291* | 10.01 | (CGT)4 | [CAAGTCGTGATCATGCGTAGGTAG](https://www.maizegdb.org/data_center/primer?id=246304) | [ACTGCTCCAGGGTGAACTGAAC](https://www.maizegdb.org/data_center/primer?id=246305) |
| 308 | *umc2034* | 10.02 | (GCTA)5 | [TATCTCCTCCGATCCTAACACCCT](https://www.maizegdb.org/data_center/primer?id=309078) | [GCTCATACGGAGGGTCAGCTAAG](https://www.maizegdb.org/data_center/primer?id=309079) |
| 309 | *phi052* | 10.02 | AAG | [CAGAATGGGACGACAAGGTCATC](https://www.maizegdb.org/data_center/primer?id=130841) | [GGGACACTTCTAGCAGGATCTGTTT](https://www.maizegdb.org/data_center/primer?id=130842) |
| 310 | *phi063* | 10.02 | TATC | [GGCGGCGGTGCTGGTAG](https://www.maizegdb.org/data_center/primer?id=111691) | [CAGCTAGCCGCTAGATATACGCT](https://www.maizegdb.org/data_center/primer?id=111692) |
| 311 | *umc1866* | 10.03 | (AT)7 | [AAGTTGGAATAATTCGGGCAGTTC](https://www.maizegdb.org/data_center/primer?id=301639) | [GGTAGCAGTAGGAGCAGAGGTGTG](https://www.maizegdb.org/data_center/primer?id=301640) |
| 312 | *umc1367* | 10.03 | (CGA)6 | [TGGACGATCTGCTTCTTCAGG](https://www.maizegdb.org/data_center/primer?id=248700) | [GAAGGCTTCTTCCTCGAGTAGGTC](https://www.maizegdb.org/data_center/primer?id=248701) |
| 313 | *umc1115* | 10.04 | (AG)6 | [TGGAAGGGGATATCAGGATTTAGA](https://www.maizegdb.org/data_center/primer?id=235070) | [TGTGATGACCATGAATGTAAGCTG](https://www.maizegdb.org/data_center/primer?id=235071) |
| 314 | *umc1077* | 10.04 | (CA)15(CGCA)12 | [CAGCCACAGTGAGGCACATC](https://www.maizegdb.org/data_center/primer?id=194000) | [CAGAGACTCTCCATTATCCCTCCA](https://www.maizegdb.org/data_center/primer?id=194001) |
| 315 | *umc1697* | 10.04 | (CGA)4 | [CAACACGTACGAAGCGCAGTC](https://www.maizegdb.org/data_center/primer?id=292438) | [TGCAGCTACCAAGTTAGCAGGAAC](https://www.maizegdb.org/data_center/primer?id=292439) |
| 316 | *umc1677* | 10.05 | (GGC)4 | [TGCAGCAAGTTTGGCTACTGC](https://www.maizegdb.org/data_center/primer?id=291364) | [CTCTTGATGCCGTTGAAGAAGG](https://www.maizegdb.org/data_center/primer?id=291365) |
| 317 | *umc1045* | 10.05 | GGA3GAG5 | [GCTCGTCCATGAGCAGCATC](https://www.maizegdb.org/data_center/primer?id=193907) | [AAGCTGAAGATGCGGAGGTTG](https://www.maizegdb.org/data_center/primer?id=193908) |
| 318 | *bnlg2190* | 10.06 | AG(31) | [TCCTCCTTCATCCCCTTCTT](https://www.maizegdb.org/data_center/primer?id=171604) | [CCCAGTATCATTGCCCAATC](https://www.maizegdb.org/data_center/primer?id=171605) |
| 319 | *umc1196a* | 10.07 | CACACG | CGTGCTACTACTGCTACAAAGCGA | AGTCGTTCGTGTCTTCCGAAACT |
| 320 | *umc2021* | 10.07 | (TGG)4 | [AAACTCAAGCTCGGAATGTACTGC](https://www.maizegdb.org/data_center/primer?id=309039) | [CGATACTGATCTACTTCACGCTGG](https://www.maizegdb.org/data_center/primer?id=309040) |
| 321 | *bnlg1677* | 10.07 | AG(36) | [GAGCAGAGCAGCTCCAAGAT](https://www.maizegdb.org/data_center/primer?id=171418) | [AACAAGACGGGAGACAATGG](https://www.maizegdb.org/data_center/primer?id=171419) |
| 322 | *umc1084* | 10.07 | (CT)23 | [GATAAAAAGGCAAGTGCAACAAGG](https://www.maizegdb.org/data_center/primer?id=194021) | [ATATCAACCAGAGGCTGGAACTTG](https://www.maizegdb.org/data_center/primer?id=194022) |
| 323 | *bnlg1450* | 10.07 | AG(34) | [ACAGCTCTTCTTGGCATCGT](https://www.maizegdb.org/data_center/primer?id=171308) | [GACTTTGCTGGTCAGCTGGT](https://www.maizegdb.org/data_center/primer?id=171309) |
| 324 | *umc2126* | 10.07 | (CGC)6 | [CAGTTCTGCACTTCTGCTTGCTC](https://www.maizegdb.org/data_center/primer?id=484972) | [AGGACTGTGAAGAGCGCGAG](https://www.maizegdb.org/data_center/primer?id=484973) |

**
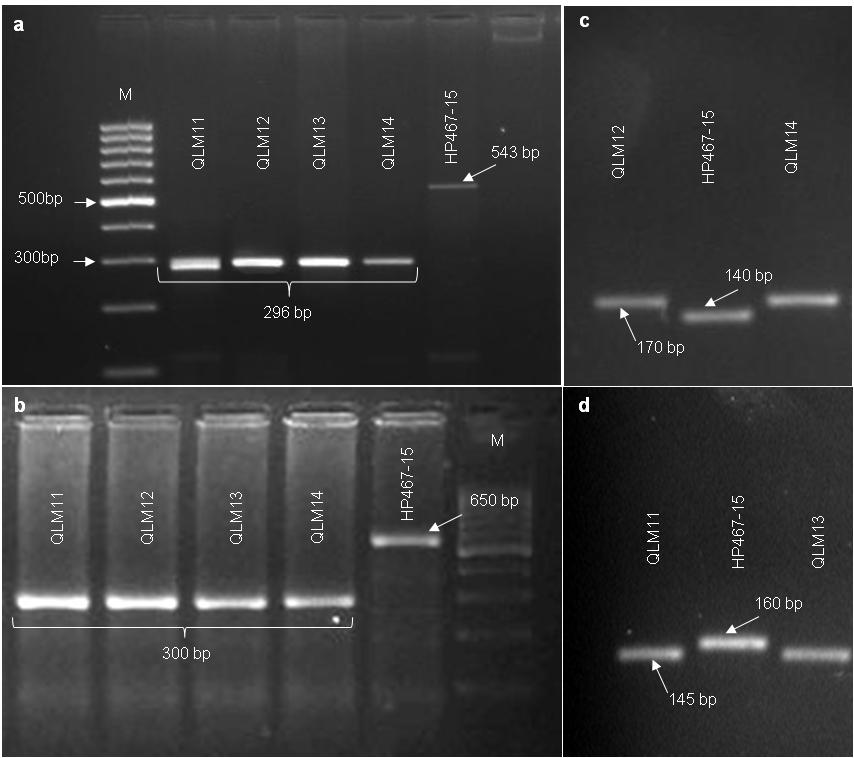
**

**Supplementary Fig. S1** Validation of gene-based markers between recurrent parents and donor parent. **(a),** PCR profile of favorable alleles for *crtRB1* gene using *crtRB1* 3ˊTE InDel marker; **(b),** PCR profile of favorable alleles for *lcyE* gene using *lcyE* 5ˊTE InDel marker; **(c),** Amplification profile for selection of *o2* gene using *phi057* SSR marker; **(d),** Amplification profile for selection of *o2* gene using *umc1066* SSR marker.

**
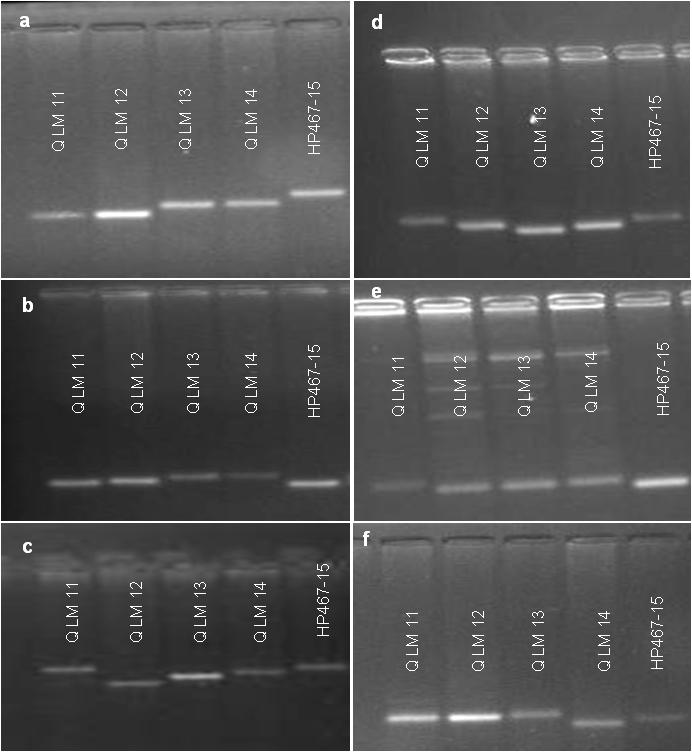
**

**Supplementary Fig. S2** Parental polymorphism survey between recurrent parents (QLM11, QLM12, QLM13, and QLM14) and donor parent (HP467-15) for background selection using SSR markers.

**(a),** bnlg381; **(b)**, umc1030; **(c)**, umc1793; **(d)**, bnlg 1887; **(e)**, bnlg1081; **(f)**, bnlg1080.

**
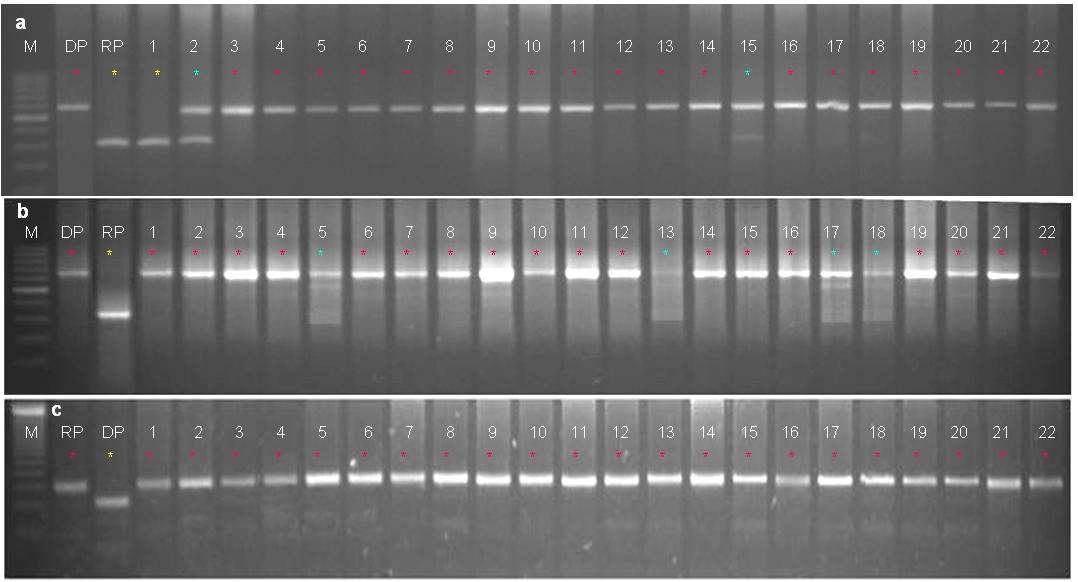
**

**Supplementary Fig. S3** Confirmation of presence of genes in homozygous condition in BC_2_F_3_ progenies of cross QLM14 × HP467-15//QLM14. **(a),** amplification profile for selection of *crtRB1* gene using *crtRB1 3ˊTE* marker; **(b),** amplification profile for selection of *lcyE* gene using *lcyE 5ˊTE* marker; **(c)**, amplification profile for selection of *o2* gene using *phi057* marker. RP: recurrent parent (QLM14); DP: donor parent (HP467-15); M: 100 bp DNA ladder; Red star indicates the desirable favorable alleles in homozygous condition; Yellow star indicates homozygous alleles to recurrent parent; Green star indicates both alleles present in heterozygous state. The progenies with number 1, 2, 5, 13, 15, 17 and 18 were rejected on the basis of marker analysis.
